# Development of Stimuli-Responsive Polymeric Nanomedicines in Hypoxic Tumors and Their Therapeutic Promise in Oral Cancer

**DOI:** 10.3390/polym17081010

**Published:** 2025-04-09

**Authors:** Jialong Hou, Zhijun Xue, Yao Chen, Jisen Li, Xin Yue, Ying Zhang, Jing Gao, Yonghong Hao, Jing Shen

**Affiliations:** 1 Department of Operative Dentistry and Endodontics, Tianjin Stomatological Hospital, School of Medicine, Nankai University, Tianjin 300041, China; houjialong19881219@163.com (J.H.); 18561515851@163.com (Z.X.);; 2 Tianjin Key Laboratory of Oral and Maxillofacial Function Reconstruction, Tianjin 300041, China; 3 Tianjin Key Laboratory for Disaster Medicine Technology, Institution of Disaster and Emergency Medicine, Tianjin University, Tianjin 300072, China; lijisen1@163.com; 4 Department of International VIP Dental Clinic, Tianjin Stomatological Hospital, School of Medicine, Nankai University, Tianjin 300041, China; 5 The Second Clinical Division, Tianjin Stomatological Hospital, School of Medicine, Nankai University, Tianjin 300041, China

**Keywords:** polymeric nanomedicines, stimulus response, hypoxic tumors, oral cancer

## Abstract

Hypoxic tumors pose considerable obstacles to cancer treatment, as diminished oxygen levels can impair drug effectiveness and heighten therapeutic resistance. Oral cancer, a prevalent malignancy, encounters specific challenges owing to its intricate anatomical structure and the technical difficulties in achieving complete resection, thereby often restricting treatment efficacy. The impact of hypoxia is particularly critical in influencing both the treatment response and prognosis of oral cancers. This article summarizes and examines the potential of polymer nanomedicines to address these challenges. By engineering nanomedicines that specifically react to the hypoxic tumor microenvironment, these pharmaceuticals can markedly enhance targeting precision and therapeutic effectiveness. Polymer nanomedicines enhance therapeutic efficacy while reducing side effects by hypoxia-targeted accumulation. The article emphasizes that these nanomedicines can overcome the drug resistance frequently observed in hypoxic tumors by improving the delivery and bioavailability of anticancer agents. Furthermore, this review elucidates the design and application of polymer nanomedicines for treating hypoxic tumors, highlighting their transformative potential in cancer therapy. Finally, this article gives an outlook on stimuli-responsive polymeric nanomedicines in the treatment of oral cancer.

## 1. Introduction

### 1.1. Background

#### 1.1.1. Mechanisms of Hypoxic Tumor Formation and Their Implications for Treatment

Hypoxic tumors are characterized by a localized hypoxic environment resulting from insufficient oxygen availability in certain regions [1]. Rapid tumor growth leads to disorganized vasculature that fails to meet tissue oxygen and nutrient demands. This aberrant angiogenesis is further aggravated by compromised tumor oxygenation resulting from structurally defective blood vessels (e.g., leaky architecture and impaired perfusion) [2]. Additionally, the elevated metabolic requirements and accelerated proliferation of tumor cells intensify oxygen utilization, while heightened anaerobic metabolism in hypoxic conditions results in lactic acid buildup and lowers the pH of the tumor microenvironment. This acidic environment exacerbates hypoxia and facilitates the adaptation and invasive potential of tumor cells under hypoxic conditions [3].

Hypoxia results from accelerated tumor proliferation and serves as a crucial mechanism for adaptation to a detrimental microenvironment, significantly influencing cancer therapies, particularly radiotherapy, chemotherapy, immunotherapy, and targeted therapy. Radiotherapy depends on the production of free radicals from oxygen to damage tumor cell DNA, while hypoxia restricts free radical generation, rendering tumor cells less responsive to radiation and diminishing the effectiveness of radiotherapy [4]. The cytotoxic effects of chemotherapeutic drugs also require the participation of oxygen. Hypoxia reduces the sensitivity of tumor cells to these drugs and further weakens the effect of chemotherapy through the activation of autophagy and anti-apoptotic mechanisms [5]. In immunotherapy, hypoxia diminishes the functionality of NK cells and T cells while facilitating the accumulation of immunosuppressive cells, thereby creating an immunosuppressive microenvironment that significantly hinders the efficacy of immunotherapy [6]. In addition, hypoxia activates the hypoxia-inducible factor (HIF) pathway, causing tumor cells to become resistant to targeted therapies such as angiogenesis inhibitors and altering tumor metabolism, thus reducing the efficacy of targeted drugs [7]. More importantly, Hypoxia further increases the invasive and metastatic potential of tumor cells, while simultaneously stimulating tumor growth through the activation of pathways linked to matrix metalloproteinases (MMPs) and vascular endothelial growth factors (VEGFs) [8].

Clinical practice has shown that high-dose chemotherapy not only fails to address the above problems but also increases the systemic toxicity of chemotherapeutic drugs. Consequently, the presence of hypoxia has been shown to reduce the effectiveness of various therapeutic interventions, promote tumor progression and metastasis, and thereby present a significant challenge to effective cancer treatment.

#### 1.1.2. Stimuli-Responsive Polymeric Nanomedicines and Their Potential in Tumor Therapy

Polymer nanodrugs are polymer-based nanoparticles loaded with drugs via nanotechnology. These nanodrugs are designed to provide innovative strategies for controlled drug release, targeted therapy, and other applications. The research directions of particular significance for polymer nanomedicines include enhancing drug efficacy and reducing toxic side effects during treatment. Specifically, stimuli-responsive polymeric nanomedicines have emerged as a pivotal tool for the precision treatment of hypoxic tumors due to their unique characteristics in tumor therapy [9].

Critical to their design is the integration of both stimulus sensitivity and biodegradability. While the primary focus of this section remains on stimulus–response mechanisms, it is noteworthy that certain biodegradable polymers (e.g., hyaluronic acid) inherently exhibit tumor microenvironment responsiveness through enzymatic degradation pathways.

Stimuli-responsive polymeric nanomedicines enable spatiotemporally controlled drug release in response to specific stimuli. Moreover, nanomedicines that have been modified with targeting ligand-functionalized exhibit a high degree of selectivity for tumor targeting [10]. Recent advances demonstrate that biodegradability can synergize with stimulus responsiveness—for instance, the pH-triggered swelling of poly (β-amino ester) nanoparticles accelerates the hydrolytic degradation of the polymer backbone, enabling pulsatile drug release [11]. As hypoxic tumors have the physiological environment and unique biomarkers required for the above materials to respond, stimuli-responsive polymeric nanomedicine-related therapeutics have become a hot-spot for research on new approaches to hypoxic tumor-specific treatments [12].

**Scheme 1:**
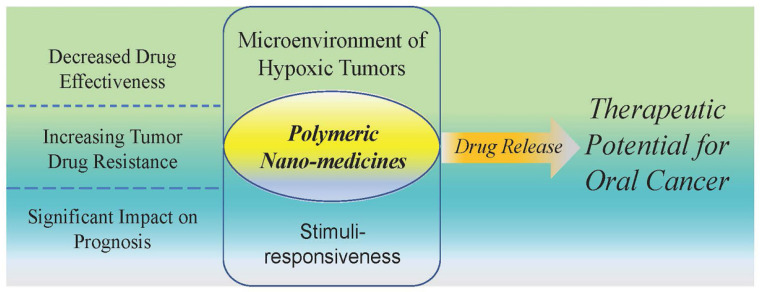
Full–text schematic diagram.

Specifically, the nanomedicines under consideration are capable of achieving a precise response in accordance with specific external or internal stimuli (e.g., pH, temperature, light, etc.). Notably, biodegradable polymers like poly(lactic-co-glycolic acid) (PLGA) have been engineered with pH-sensitive surface coatings to create dual-functional systems that combine environmental responsiveness with metabolic clearance [13]. This approach guarantees the precise delivery of the drug within hypoxic tumor microenvironments, thus optimizing therapeutic effectiveness while reducing adverse effects on surrounding healthy tissues [14] (Scheme 1).

Key innovation trends include the following:

i. Biodegradable polymer backbones (e.g., chitosan) functionalized with stimulus-sensitive moieties (e.g., sulfonamide groups for pH responsiveness)
ii. Enzyme-cleavable linkages in natural polymers (e.g., hyaluronidase-degradable hyaluronic acid carriers)

By leveraging the distinctive characteristics of the tumor microenvironment (e.g., overexpressed enzymes or particular membrane proteins) and integrating them with surface-modified targeting agents, these pharmaceuticals can attach to specific receptors on tumor cells, facilitating highly selective and precise delivery while markedly minimizing off-target effects. Moreover, their nanoscale dimensions facilitate effective accumulation at the tumor site through enhanced permeability and retention (EPR) effects, while their substantial drug-loading capacity guarantees adequate local concentration to augment therapeutic efficacy [15].

Biodegradable, encompassing both natural materials (e.g., chitosan, hyaluronic acid, and gelatin) and synthetic counterparts (e.g., polylactic acid (PLA) and polyethylene glycol (PEG)), have emerged as pivotal raw materials for designing polymeric nanomedicines. In contrast to lipid-based systems, which may face issues like instability and limited drug-loading capacity, polymeric nanomedicines offer enhanced stability and a higher drug payload, making them more suitable for targeted delivery in complex tumor microenvironments [16]. Natural polymers are distinguished by their superior biocompatibility, biodegradability, and tumor-targeting capabilities facilitated by the enhanced permeability and retention (EPR) effect. These biomaterials can be chemically functionalized to optimize performance; for instance, hyaluronic acid-based nanocarriers enable precise drug release within the tumor microenvironment [17,18]. Synthetic polymers, on the other hand, offer tunable mechanical strength, controllable degradation rates, and structural precision, making them ideal for engineering stable, multifunctional platforms that integrate tumor imaging, diagnostics, and combination therapy [19]. Such platforms allow the real-time monitoring of drug delivery and therapeutic response through the incorporation of imaging agents and therapeutic molecules. However, synthetic polymers face challenges in clinical translation due to potential immunogenicity, whereas natural polymers may suffer from batch-to-batch variability. Consequently, the selection of optimal nanocarriers requires balancing material properties (e.g., biocompatibility and stability), disease-specific demands, and patient heterogeneity to maximize therapeutic efficacy while minimizing adverse effects [20].

**Figure 1:**
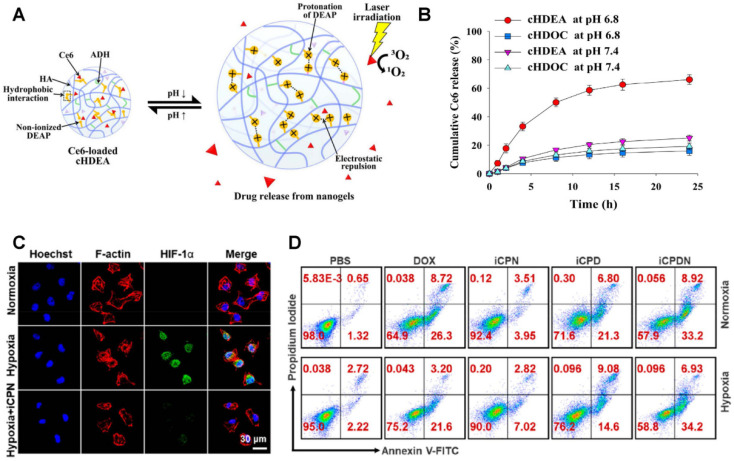
(**A**) Schematic illustration of Ce6-loaded cHDEA nanogels [21], Copyright 2023, Wiley; (**B**) cumulative release profile of Ce6 from the nanogels at pH 7.4 or pH 6.8 over a 24 h period [21], Copyright 2023, Wiley; (**C**) immunofluorescence analysis of HIF-1α level in 4T1 cells after different treatments [22], Copyright 2022, American Chemical Society; (**D**) flow cytometry analysis of apoptosis in 4T1 cells treated with PBS, DOX, iCPN, iCPN, or iCPDN [22], Copyright 2022, American Chemical Society.

**Figure 2:**
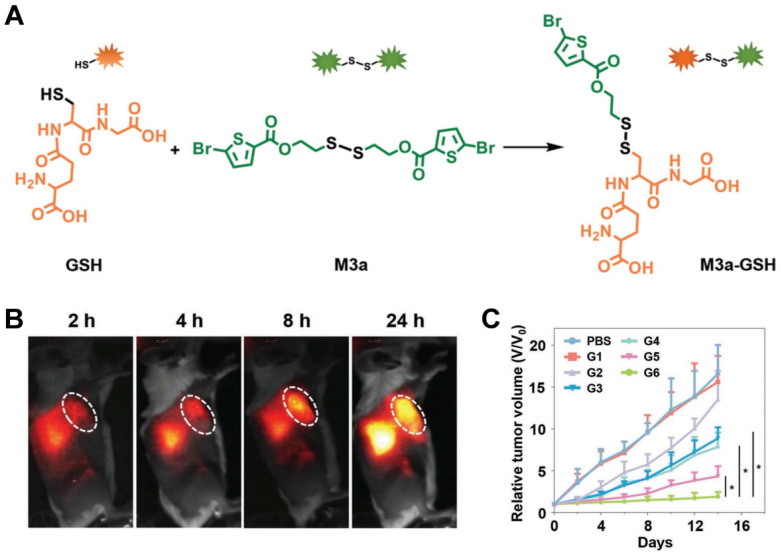
(**A**) Schematic illustration of the possible reaction between M3a and GSH [23], Copyright 2022, Wiley; (**B**) NIR-II fluorescence bioimaging of mice injected with NP@PEDOX/PSP after various time points in vivo [23], Copyright 2022, Wiley; (**C**) the relative tumor volume change curve during treatment. (* *p* < 0.05) Copyright 2022, Wiley [23]. Copyright 2022, Wiley.

For example, in 2023, Jeonghun Song et al. developed hyaluronic acid (HA)-based pH-dependent swellable nanogels (Figure 1A,B) for acidic tumors [21]. This system uniquely combines HA’s inherent biodegradability via hyaluronidase with pH-triggered swelling behavior, achieving both targeted degradation and stimulus-responsive drug release. Through the meticulous modulation of drug release via pH, these nanomedicines successfully sustained elevated drug concentrations within the tumor area, exhibiting a drug release rate three-fold greater at pH 6.8 than at pH 7.4, alongside enhanced cellular uptake of the drug at pH 6.8 relative to pH 7.4. Wang K et al. The group developed a nanosized system that circumvents the resistance mechanisms of healthy tissues and surmounts the resistance of hypoxic tumors to conventional chemotherapy (Figure 1C,D and Table 1) [22]. The preparation was achieved using the tumor-acidic reactive moiety poly (2-azirane ethyl methacrylate), which has a fast reaction rate and efficient bioorthogonal click chemistry. This polymer forms large aggregates in tumor tissue, enhancing accumulation and retention. Subsequently, the tumor-acidic reactive moiety of another slower-reacting maleic acid amide was cleaved, allowing the aggregates to slowly dissociate into ultra-small nanoparticles with improved tumor penetration. The study delivered doxorubicin (DOX) and nitric oxide (NO) to hypoxic tumor tissue. In addition, the systemic delivery of NO significantly downregulated HIF-1α levels, reversed hypoxia-induced DOX resistance, and enhanced anti-tumor immune responses by reprogramming the tumor immune microenvironment. At pH 6.5, the cumulative drug release over 48 h was five times greater than that at pH 7.4, and the concurrent administration of immunotherapy significantly enhanced both the concentration and retention of the drug within tumor tissues in the experimental group. The aforementioned strategies effectively produced synergistic effects, enhancing overall therapeutic outcomes and mitigating the hypoxia-induced chemoresistance in conventional treatment methods. Tang D’s group additionally discovered that certain polymer nanoparticles (Figure 2) [23] have potential for both tumor imaging and diagnostic applications. Through NIR light excitation, the polymer nanoparticles could effectively generate ROS (reactive oxygen species) and induce the rapid release of DOX, and at the same time exhibit strong NIR-II fluorescence, and the fluorescence signals in the tumor were 1.19- and 1.27-fold stronger than those in the kidney and lung, respectively, indicating that the polymer nanoparticles have good tumor-targeting effects. The proportion of mature antigen-presenting cells activated by polymer nanoparticles was 3.3-fold higher than that of the control group. At the same time, NP@PEDOX/PSP significantly restrained the growth of cancer cells when subjected to light irradiation in vitro. Moreover, in vivo studies demonstrated that NP@PEDOX/PSP provided remarkable near-infrared II (NIR-II) fluorescence signals suitable for bioimaging applications in the NIR-II spectrum. NP@PEDOX/PSP under photoexposure effectively inhibited 4T1 tumor growth without significant side effects. In addition, light-exposed NP@PEDOX/PSP-treated mice recruited dendritic cells, promoted antigen-specific cytotoxicity, and induced T lymphocytes to the tumor microenvironment. This outstanding research emphasizes the utilization of self-decomposing degradable PSP in NIR-II fluorescence bioimaging, photodynamic immunotherapy, and photoacoustic computed tomography (PACT) for cancer treatment.

Nanoparticles can facilitate fluorescent bioimaging, photodynamic immunotherapy, and photoactivated chemotherapy, thereby enabling imaging-guided monitoring for precision therapy and offering a novel multimodal therapeutic approach for cancer treatment. The adaptability of stimuli-responsive polymeric nanomedicines renders them a promising and effective strategy in tumor therapy. As research advances, we anticipate that stimuli-responsive polymeric nanomedicines will significantly enhance cancer treatment and improve patient outcomes.

Despite the significant advantages of nanomaterials in drug delivery, their stability issues cannot be overlooked. The high surface energy of nanomaterials endows them with dual characteristics of low stability and high reactivity, which may lead to oxidation or degradation during application, thereby compromising their functionality and therapeutic efficacy [50]. Therefore, investigating and optimizing the stability of nanomaterials is critical for advancing their applications in drug delivery systems.

### 1.2. Specificities of Oral Cancer

#### 1.2.1. Oral Cancer and Clinical Treatment Challenges

Oral cancer is one of the most common malignant tumors worldwide, with a particularly high incidence in Asia, Southeast Asia, India, and the Middle East [51]. According to the World Health Organization (WHO), there are nearly 300,000 new cases reported each year, positioning it among the primary contributors to mortality associated with cancer. The primary risk factors for oral cancer include tobacco use, alcohol intake, human papillomavirus infection, inadequate oral hygiene, poor dietary practices, genetic susceptibility, immunocompromised states, and prolonged ultraviolet light exposure [52].

The early symptoms of oral cancer are often subtle, leading to frequent diagnosis at an advanced stage. Delayed diagnosis increases the likelihood of cancer spread, worsening treatment outcomes and prognosis [53]. Moreover, oral cancer exhibits high local invasiveness, especially in the tongue and floor of the mouth, complicating surgical resection, while its high rate of lymphatic metastasis, especially in the cervical lymph nodes, has a significant impact on survival [54]. Treatment typically necessitates a multidisciplinary strategy encompassing surgery, radiotherapy, chemotherapy, targeted therapy, and immunotherapy; however, each modality has limitations such as surgical trauma, side effects from radiotherapy, and resistance to chemotherapy [55]. Patients often face post-treatment challenges, including speech, swallowing, and respiratory impairments due to treatment, particularly following significant tissue excision. The surgical recovery phase necessitates rehabilitation of speech, swallowing, and chewing functions, making oral reconstruction and rehabilitation critical to postoperative quality of life [56]. However, delayed diagnosis, local aggressiveness, metastasis, and complex multidisciplinary treatment demands render oral cancer management challenging. Therefore, the development of innovative diagnostic and therapeutic strategies is crucial to improve patient survival.

#### 1.2.2. Treatment of Oral Cancer and the Anoxic Environment

The hypoxic microenvironment significantly influences oral cancer treatment by enhancing tumor cell drug resistance, diminishing radiotherapy efficacy, impairing drug delivery, and facilitating immune evasion [57].

Primarily, early diagnosis is crucial for enhancing the cure rate of oral cancer; however, a hypoxic environment may complicate early tumor detection. Cells in hypoxic tumor regions frequently exhibit dormancy or reduced proliferation rates, and preoperative MRI and CT examinations may inadequately detect early oral tumors due to limited resolution, which complicates the accurate delineation of the true extent of oral tumors, particularly those located deep within the oral cavity, during early imaging [58].

The hypoxic environment also poses significant challenges to the treatment of oral cancer. The hypoxic environment leads to the deterioration of the therapeutic effects of chemotherapeutic and immunological drugs on oral tumors, and more importantly, the treatment of oral cancer is not only concerned with the removal of tumors, but also the restoration of postoperative function. However, the hypoxic environment may lead to a wider range of surgical procedures for oral cancers through mechanisms that promote tumor invasiveness, potential metastasis, border-blurring, and enhanced tolerance [59].

To guarantee total excision of the tumor postoperatively and mitigate the risk of recurrence, the surgical procedure may necessitate the inclusion of a broader expanse of healthy tissue. This not only elevates the complexity of the procedure but may also influence the patient’s postoperative function and quality of life, significantly impacting postoperative recovery [60].

Therapeutic strategies for hypoxic environments are gradually becoming a research priority in oral cancer treatment. In order to overcome the therapeutic challenges of hypoxic tumors, researchers have proposed a variety of innovative therapeutic approaches, such as the development of hypoxia-activated drugs [61] and enhanced nanomedicine targeting [62]. With the deepening understanding of the hypoxic tumor microenvironment advances, novel therapeutic alternatives are being formulated, offering a broader array of options for oral cancer treatment.

In summary, the hypoxic environment is a pivotal factor in the development, progression, and management of oral cancer. Novel therapeutic approaches, such as targeted treatments, nanomedicine delivery systems, and oxygen-enhanced therapies tailored for hypoxic microenvironments, present significant potential in addressing the difficulties encountered in the management of hypoxic tumors. These strategies are expected to enhance the therapeutic efficacy of oral cancer and provide new solutions to the prognostic challenges faced by oral cancer patients.

This paper undertakes a comprehensive analysis of the current state of research on stimuli-responsive polymeric nanomedicines in the treatment of hypoxic tumors, with a particular focus on their potential application in the management of oral cancer. The core role and development of stimuli-responsive polymer nanomedicines in the treatment of hypoxic tumors are systematically described from the perspectives of hypoxic tumor formation mechanisms and advantages of polymer nanomedicines, the unique features of oral cancers and key points of nanomedicine design, typical cases and potential applications in oral cancers, existing problems and challenges, and conclusions and future directions. This study provides a comprehensive reference for the development of drug therapies and clinical practices for the treatment of hypoxic tumors, with a particular focus on oral cancer.

## 2. Design Concepts for Stimuli-Responsive Polymer Nanomedicines for Hypoxic Tumors

### 2.1. Introduction to Amphiphilic Polymers

Amphiphilic polymers, which contain both hydrophilic and hydrophobic segments, are frequently utilized as nanocarriers and constitute essential components of nanomedicines. Due to their distinctive structural characteristics, these polymers possess the capacity to spontaneously assemble into stable nanostructures within an aqueous environment through intermolecular hydrophilic–hydrophobic interactions. The core of these nanostructures is formed by hydrophobic segments, which encapsulate the hydrophobic drug, while the hydrophilic segments form an outer protective shell to increase the water-solubility and stability of the carrier. The simplicity and efficiency of the self-assembly process, as well as the ease of scale-up production, make amphiphilic copolymers an important material for the development of nanodrug carriers. Through precise modulation of the hydrophilic and hydrophobic group ratios, molecular weight, and structural design, the size, morphology, and drug-loading capacity of these carriers can be meticulously tailored to enhance their stability and optimize their in vivo distribution behavior. While inorganic nanomaterials (e.g., magnetic or silica-based nanoparticles) demonstrate potential in targeted drug delivery due to their stimulus-responsive properties and high mechanical strength [63], amphiphilic polymers still maintain their dominance in the development of intelligent drug delivery systems [64]. Their advantages lie in high molecular tunability, controllable metabolic pathways, and biomimetic compatibility. For instance, in contrast to inorganic carriers that rely on external magnetic or photothermal manipulation, amphiphilic polymers can achieve precise microenvironment-triggered drug release through pH- or enzyme-responsive functional groups [65]. This strategy offers a more universal solution for treating diseases such as cancer by enabling context-specific therapeutic activation within pathological tissues.

Furthermore, the delivery and release mechanisms of nanomedicines are predominantly contingent on the structural design and responsive properties of the nanocarriers. Through the modification of the structure and functionalization of amphiphilic polymers, the release and targeted delivery of drugs can be effectively regulated. During the delivery process, the hydrophobic core effectively encapsulates the hydrophobic drug, while the hydrophilic shell provides stability and prevents premature release or degradation of the drug. The hydrophilic portion of the shell can bind targeting ligands (e.g., antibodies, short peptides, and other functional molecules), enabling the carrier to accurately identify and accumulate in specific regions, such as blood vessels and inflammatory sites in hypoxic tumors. This results in a significant increase in the concentration of the drug in the tumor area and a reduction in its side effects on healthy tissues. This design establishes the foundation for achieving stimuli-responsive and precise drug delivery to hypoxic tumors.

When designing nanocarriers, considerations must extend beyond drug-loading capacity and targeting specificity to encompass their structural stability. The stability of nanomaterials critically influences not only their circulatory half-life in the bloodstream but also their controlled release and therapeutic efficacy within tumor microenvironments. Stability can be significantly enhanced through alloying strategies, grain boundary engineering, or morphological control. For instance, the incorporation of stabilizing agents or surface modifications has been shown to improve the thermal and chemical stability of nanoparticles, thereby improving their potential for precision drug delivery applications [66].

### 2.2. Targeting of Hypoxic Tumor-Related Nanomedicines

#### 2.2.1. Passive Targeting of Nanomedicines

The vascular architecture of tumors is typically irregular and highly permeable, facilitating the ingress of nanoparticles into tumor tissue via the tumor vasculature and their retention within the tumor due to inadequate lymphatic drainage. This phenomenon enhances the local concentration of the drug, and the tumor-specific enhanced permeability and retention (EPR) effect serves as a crucial passive targeting strategy in nanomedicine research. The physicochemical characteristics of polymeric nanocarriers, including optimal particle size (<400 nm) and hydrophilic surface properties, facilitate increased drug accumulation in tumors. Hydrophilic surfaces can further reduce non-specific protein adsorption and prolong systemic circulation, thereby enhancing tumor-specific accumulation through the EPR effect [67].

For example, Popov’s study describes the design of a novel immunocompatible polymer nanocarrier F127@PDA made from melanin-mimicking polydopamine and Pluronic F127 units. The nanocarriers were synthesized through a robust and reproducible technique that was modified to yield a variety of particle dimensions (less than 100 nm) while maintaining the original composition of the carriers. A comprehensive series of in vitro experiments were conducted to evaluate the influence of carrier size (40, 60, and 100 nm) on the immunocompatibility, cell viability, and uptake by distinct pancreatic cancer cell lines that exhibit diverse morphological and phenotypic traits. The study found that drug-loaded nanocarriers with three different particle sizes had more significant anti-proliferative effects on all tumor cell lines than free drug [68]. Bo Yu et al. employed hydrophilic polylysine (PLL) as a delivery vehicle to fabricate drug-loaded nanoparticles using the hydrophobic drug methotrexate (MTX), which formed stable nanoparticles via electrostatic interactions between the carboxyl groups of methotrexate and the amine groups of PLL. The hydrophilic surface of PLL/MTX nanoparticles demonstrated superior oncological efficacy relative to conventional nanomedicines, exhibiting a 1.7-fold enhancement in tumor cell inhibition compared to PEG-PLL/MTX nanoparticles. Mechanistically, the hydrophilic PLL surface reduced opsonization and macrophage clearance, allowing more nanoparticles to extravasate into tumor tissues [69]. Animal studies have also demonstrated that hydrophilic surfaces can augment the anti-tumor efficacy of drugs, resulting in a 1.2-fold increase in the tumor inhibition rate of PLL/MTX nanoparticles compared to conventional surface polymer drugs [70]. The hydrophilic characteristics of surfaces can be utilized to develop nanoscale drug delivery systems that exhibit improved anticancer efficacy.

#### 2.2.2. Active Targeting Based on the Hypoxia-Associated Proteins

In hypoxic tumor therapy, polymeric nanomedicines can effectively enhance the therapeutic effect by targeting tumor-specific hypoxia-associated proteins. Hypoxia-inducible factors (HIFs) are key factors for tumors to regulate adaptation in a hypoxic environment, and targeting HIF-1α and HIF-2α can inhibit the adaptive response of tumor cells and prevent tumor growth and metastasis. For example, triple-negative breast cancer (TNBC) lacks targeted therapy and has a poor prognosis, and the tumor microenvironment (TME) hinders chemotherapy. Xuemeng Liu et al. investigated the preparation of hypoxia-responsive polymeric micelles co-loaded with adriamycin (DOX) and shRNA targeting HIF-1α (shHIF-1α) for the synergistic treatment of TNBC. Negatively charged shHIF-1α electrostatically binds to the azo-PM micelles’ surface to prepare azo-PM/DOX+shHIF-1α. Under hypoxic conditions, the azo undergoes bioreductive cleavage, leading to the separation of hydrophilic and hydrophobic segments of the copolymers, and the subsequent disintegration of assembled micelles, which releases the payload through targeting the tumor hypoxia and HIF-1α pathway, showing good synergistic therapeutic efficacy and safety in TNBC treatment, presenting an innovative approach for the management of triple-negative breast cancer (TNBC) and other tumors characterized by hypoxia [71]. In addition, polymeric nanomedicines possess the capability to selectively target vascular endothelial growth factors (VEGFs) along with its receptor, VEGFR-2, facilitating both imaging and therapeutic applications. VEGF is overexpressed in tumor angiogenesis and existing antibody-targeted therapies are deficient. Molecularly imprinted polymer nanoparticles (MIPs) have advantages in terms of stability, speed of design, cost, and control of functionalization. This study aims to prepare molecularly imprinted polymer nanoparticles targeting VEGF and to explore their application in cancer imaging. In this study, nine amino acid epitopes (amino acids 83–91: IKPHQGQHI) on the surface of synthesized hVEGF were selected as templates for the solid-phase synthesis of molecularly imprinted polymeric nanoparticles (MIPs) against hVEGF. Quantum dots (QDs) were covalently coupled to the surface of the MIPs to achieve in vivo fluorescence imaging. The QD-MIPs have demonstrated good performances in both in vitro and in vivo experiments, and are expected to be a viable alternative to traditional antibodies and a new tool for cancer research [72].

Polymeric nanomedicines can also target proteins related to tumor metabolism and survival, such as carbonic anhydrase IX (CAIX) [73], to disrupt the acidic environment and energy supply of tumors and further inhibit tumor growth. Meanwhile, targeting anti-apoptotic proteins (e.g., Bcl-2 [74], Bcl-xL [75]) can promote the apoptosis of tumor cells and improve the therapeutic effects. In addition, targeting resistance-associated proteins (e.g., P-glycoprotein [76]) helps reverse chemotherapy resistance in tumors.

### 2.3. Microenvironmental Responses Associated with Hypoxic Tumors

The microenvironment of hypoxic tumors is characterized by low oxygen levels, acidity, and a high lactate concentration, which limit the efficacy of conventional therapeutic approaches. To address these issues, scientists have created polymeric nanomedicines that are responsive to the tumor microenvironment, facilitating targeted drug release at the tumor site triggered by alterations in the microenvironment (such as hypoxia and acidity). This innovative approach enhances the effectiveness of therapies while minimizing adverse effects.

#### 2.3.1. Tumor-Related pH Response to Hypoxia

The acidic characteristics of the tumor microenvironment cause drug carriers to undergo protonation or deprotonation reactions, resulting in accelerated drug release. Conversely, normal tissues exhibit a higher pH, resulting in diminished drug release in these areas, thereby mitigating toxic effects on healthy cells and facilitating more targeted treatment.

The acidic microenvironment of hypoxic tumors provides distinct benefits for the development of pH-responsive polymeric nanodrugs. These drug delivery systems generally consist of polymers featuring pH-sensitive groups, such as poly (lactic acid-glycolic acid) copolymer (PLGA) and poly(N-ethylacrylamide), which experience structural alterations in acidic environments, facilitating drug release. This mechanism enables efficient drug release in the tumor region, leading to increased drug concentrations and improved therapeutic efficacy. Researchers have demonstrated that pH-sensitive nanodrugs, composed of a PLGA and gelatin, can selectively release paclitaxel in the acidic microenvironment of tumors. This pH-responsive drug release mechanism guarantees that paclitaxel is predominantly released in the tumor region, minimizing its effects on normal tissues [24].

Furthermore, this delivery system is widely used to deliver anticancer drugs, including chemotherapeutic drugs like paclitaxel and cisplatin, as well as gene drugs and photosensitive materials. Polymer-based nanomedicines that respond to pH changes enhance therapeutic effectiveness while simultaneously minimizing adverse effects on healthy tissues, thereby presenting a viable and secure treatment alternative for tumors characterized by hypoxia. To improve drug delivery to tumors, researchers created a novel nanomedicine by encapsulating adriamycin in pH-sensitive microbubbles made of amphiphilic block copolymers. These microbubbles remain stable in the neutral environment of the bloodstream but release adriamycin once they enter the acidic environment of tumor tissues. The pH-responsive drug release mechanism significantly reduces adriamycin’s toxicity toward normal cells while increasing drug concentration in the tumor region and reducing its side effects on healthy tissues. Simultaneously, it increases drug concentration in the tumor area and improves therapeutic efficiency [25].

#### 2.3.2. Tumor-Associated Redox Response in Relation to Hypoxic Tumors

Another important response modality for hypoxic tumor therapy is the redox-responsive mechanism, in which polymer chains are altered under the reducing conditions of the tumor microenvironment, and the drug is rapidly released into tumor cells, allowing for precise targeting via redox-responsive nanomedicines. Polymeric nanomedicines can increase drug concentration in the tumor region while minimizing the impact on healthy tissues, significantly improving therapeutic efficacy and reducing side effects. This enables redox-responsive polymeric nanomedicines to demonstrate great promise in cancer therapy, particularly in the treatment of oxygen-depleted tumors.

**Figure 3:**
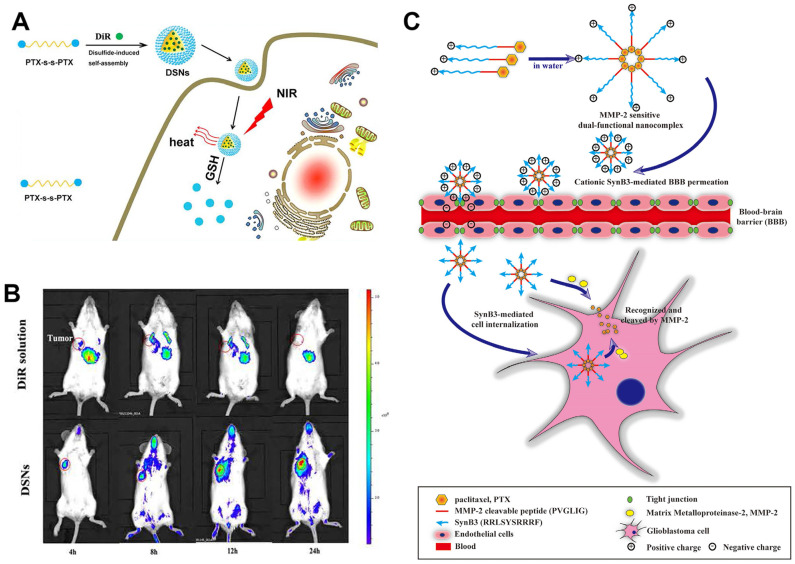
(**A**) Schematic diagram of the working principle of DSNs [27], Copyright 2016, American Chemical Society; (**B**) in vivo and ex vivo fluorescence imaging of tumor-bearing A549 mice at predetermined time intervals postinjection of DiR solution and DSNs [27], Copyright 2016, American Chemical Society; (**C**) schematic of drug delivery and release strategy of SynB3-PVGLIG-PTX [28], Copyright 2020, Wiley.

Redox-responsive polymeric nanomedicines exploit the redox imbalance characteristics prevalent in the tumor microenvironment to facilitate targeted drug delivery and release by reacting to reducing or oxidizing alterations in tumor cells or the surrounding environment. For hypoxic tumors, a more reductive environment is usually observed because tumor cells produce large amounts of reducing molecules (e.g., glutathione (GSH) under high metabolic demands. Redox-responsive polymeric nanomedicines are designed to undergo chemical changes in the reducing environment, leading to drug release in the tumor region. For example, Zhang et al. designed a redox-sensitive nanoparticle system that releases the drug in response to high GSH levels in tumor cells [77]. The system uses a disulfide bond to link the drug to the carrier, which is stable in the bloodstream but breaks in the presence of high GSH concentrations within tumor cells, releasing the drug. This mechanism ensures that the drug is released specifically in the tumor region, enhancing therapeutic efficacy and reducing side effects. Doxorubicin-loaded redox-responsive micelles composed of poly (ethylene glycol)-b-poly (dithiothiol methacrylate) (PEG-b-PDMT) are proposed to release drugs in the presence of elevated GSH levels in tumor cells. The redox sensitivity guarantees the stability of the nanomedicine during circulation, and owing to the elevated intratumoral GSH concentration, the active component can be selectively released within the tumor for targeted delivery [26]. In terms of chemical structure, this type of nanomedicine usually consists of polymers containing reversible redox-responsive groups, such as disulfide bonds, thiol groups, nitroso compounds, etc. These polymers are reduced or broken down in the reducing environment of the tumor, which in turn releases the drug. For example, Han et al. synthesized a novel PTX-s-s-PTX conjugate via disulfide bonds, achieving a high drug-loading capacity (78%, *w*/*w*) (Figure 3A,B) [27]. The adducts have the capacity to spontaneously organize into nanoparticles (NPs), within which DiR is encapsulated in the core of PTX-s-s-PTX NPs for the purpose of photothermal therapy (PTT). These DiR-loaded self-assembled nanoparticles (DSNs) demonstrated remarkable stability when subjected to biological conditions. Disulfide bonds were used as redox-responsive linkages to facilitate the rapid release of paclitaxel from tumor cells. DSN synergistic chemothermal therapy exhibits substantial cytotoxicity and exceptional efficacy, characterized by high drug-loading capacity, redox-responsive paclitaxel release behavior, photothermal agent loading capacity, and the potential for combination therapy with photothermal therapy (PTT). This efficient nanosystem integrates prodrug and photothermal strategies to augment therapeutic efficacy. This redox-sensitive method can regulate drug release, mitigate the adverse effects of traditional chemotherapy, enhance the therapeutic index, and decrease systemic toxicity.

#### 2.3.3. Enzymatic Responses Associated with a Tumor of Hypoxia

Enzyme-responsive polymeric nanomedicines are delivery systems that utilize specific enzyme activities within the tumor microenvironment for drug release, demonstrating significant potential for treating hypoxic tumors. The design of these nanomedicines relies on the elevated expression of enzymes within tumor cells or the tumor microenvironment, particularly since hypoxic tumors frequently exhibit distinct enzyme activities linked to their rapid proliferation, metastasis, and aberrant metabolism. In these enzyme-enriched environments, enzyme-responsive polymeric nanomedicines can release drugs specifically in the tumor region, enhancing therapeutic targeting and efficacy. Specific enzymes, such as matrix metalloproteinases (MMPs), are overexpressed in hypoxic tumors and can be targeted for drug release. For example, Dan et al. conjugated paclitaxel nanoparticles with an MMP-2-sensitive linker, synthesizing SynB3-PVGLIG-PTX nanodrugs that were compatible with MMP-2, a protease overexpressed in many tumors [28]. These nanodrugs showed a strong affinity for MMP-2, which enhanced water solubility and tumor-targeting efficacy, leading to significant anti-tumor activity.

Common enzymes utilized by enzyme-responsive polymeric nanomedicines include proteases (e.g., matrix metalloproteinases MMPs), peroxidases (e.g., hydrogen peroxide in tumor cells), and trypsin-like enzymes. Many hypoxic tumor cells have higher enzyme activities than normal tissues. For example, MMPs play an important role in tumor cell invasion and metastasis, and polymeric nanodrugs targeting these enzymes can trigger drug release through specific cleavage in the tumor microenvironment. Dan et al. conjugated paclitaxel nanoparticles with an MMP-2-sensitive linker, and synthesized SynB3-PVGLIG-PTX nanodrugs that were compatible with MMP-2, a protease overexpressed in many tumors. These nanodrugs showed a strong affinity for MMP-2, forming water-soluble aggregates to enhance solubility. Upon cleavage by MMP-2, SynB3-PVGLIG-PTX undergoes a controlled release of PTX, which implies that SynB3-PVGLIG-PTX has specific cytotoxicity in GBM cells. The nanodrug demonstrates significant efficacy in suppressing the proliferation, migration, and invasion of GBM cells in both in vitro and in vivo models. Its inhibition rate was significantly higher than that of temozolomide and paclitaxel. The integration of MMP-2-sensitive peptides and cell-penetrating peptide SynB3 improves the blood–brain barrier permeability and glioma-targeting efficacy of SynB3-PVGLIG-PTX, leading to significant anti-tumor activity with minimal adverse effects (Figure 3C) [28].

This strategy enables targeted drug release at tumor sites where enzymes are overexpressed, thereby minimizing the impact of the drug on healthy tissue. In addition, the high concentration of hydrogen peroxide (H_2_O_2_) in tumor cells allows enzyme-responsive polymeric nanodrugs to undergo enzyme-mediated redox reactions, triggering drug release [29].

#### 2.3.4. Light Response Associated with a Tumor of Hypoxia

Light-responsive nanomedicines facilitate the localized and precise regulation of drug release timing and location by employing light to activate drug release in a spatially controlled fashion.

**Figure 4:**
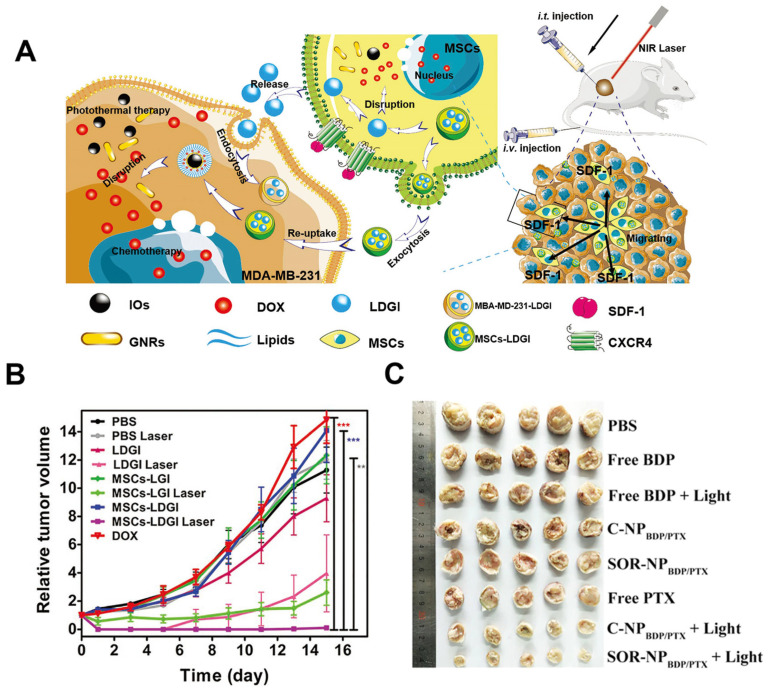
(**A**) The proposed framework for utilizing LDGI-loaded mesenchymal stem cells (MSCs) aims to augment the migratory capacity of tumors, as well as to improve the effectiveness of photothermal therapy (PTT) and chemotherapy in the treatment of triple-negative breast cancer (TNBC) [30], Copyright 2018, Wiley; (**B**) relative tumor volume of being intratumorally injected with DOX, PBS, LDGI, MSCs-LGI, and MSCs-LDGI with and without NIR laser irradiation within 15 days [30], (** *p* < 0.01, and *** *p* < 0.001) Copyright 2018, Wiley; (**C**) images of the excised tumors at the end of different treatments [31], Copyright 2021, American Chemical Society.

Light-responsive nanocarriers are engineered with nanoparticles that incorporate light-responsive linkers, such as azobenzene or coumarin derivatives. These linkers undergo chemical modifications upon light irradiation at designated wavelengths, resulting in the structural transformations of the nanoparticles that facilitate drug release [78]. Chang Xu and colleagues engineered mesenchymal stem cells (MSCs) that are incorporated with plasmonic–magnetic hybrid nanoparticles (LDGIs) for the purpose of photoacoustic imaging. This innovative approach establishes a synergistic therapeutic platform designed for targeted photothermal therapy and chemotherapy. Upon exposure to light irradiation, this system initiates concurrent drug release and photothermal treatment. Furthermore, the liberated drug is capable of permeating the cell nucleus, thereby facilitating apoptosis (Figure 4A,B) [30].

Light irradiation can regulate the secretion of cellular microcarriers with LDGI for targeted therapy. Notable in vitro anticancer effects were detected under NIR laser irradiation. In vivo studies demonstrated the enhanced migration and penetration of MSCs-LDGI in the tumor region, with a 3.33-fold increase in tumor tissue nanoparticle accumulation. MSCs-LDGI showed the best anti-tumor efficacy through chemo-photothermal therapy compared to other treatment groups, demonstrating the great synergistic potential of the MSCs-LDGI multifunctional system for the treatment of cancer. Yang D.C. et al. (Figure 4C) [31] By incorporating a ^1^O2-sensitive aminoacrylate linker into amphiphilic mPEG-*b*-PCL copolymers. A sophisticated nanocarrier for single-linear oxygen-responsive nanoparticles (SOR-NPs) was developed. Dipyrrolylmethylene boron (BDP) and paclitaxel (PTX) were co-encapsulated in ^1^O2-responsive nanocarriers for light-mediated drug release and synergistic cancer treatment. This polymeric nanocarrier effectively alleviates the aggregation of encapsulated BDP chains due to its long hydrophobic chains. The synthesized SOR-NPBDP/PTX nanodrugs exhibited intense fluorescent signals and elevated production of ^1^O2, facilitating cell death through photodynamic therapy (PDT) while concurrently degrading the aminoacrylate linker, resulting in the disintegration of SOR-NPBDP/PTX and the subsequent liberation of PTX. The light-activated drug release and synergistic anticancer effects of SOR-NPBDP/PTX were evidenced in HepG2 and MCF-7 cancer cells, as well as in H22 hormonal mice. The light-activated release mechanism ensured that the chemotherapeutic agent was administered exclusively to the targeted area, achieving elevated local concentrations while minimizing off-target adverse effects. This study presents a promising strategy for tumor-specific drug release that integrates light-responsive and reactive oxygen species-responsive delivery systems.

Furthermore, photothermal-induced drug release represents an innovative strategy for hypoxic tumor therapy that utilizes responsive polymer nanoparticles. For instance, paclitaxel-encapsulated thermo-responsive nanoparticles composed of PNIPAM release paclitaxel at increased temperatures (e.g., during thermotherapy). This temperature-activated release mechanism guarantees that the drug is released upon an increase in tumor temperature while maintaining stability and non-toxic properties during circulation at normal body temperature [79]. In addition, doxorubicin nanoparticles combined with temperature-sensitive polymers can be externally heated (using techniques such as magnetic hyperthermia) to trigger the drug’s release only at the tumor site. This local heating increases the release of doxorubicin at the tumor site, thereby increasing the therapeutic efficacy while reducing off-target effects [32].

### 2.4. Realization of Anti-Desiccated Tumor Effects

#### 2.4.1. Pharmacodynamic Optimization of Conventional Chemotherapeutic Agents

Polymeric nanodrug delivery systems enhance the therapeutic efficacy of chemotherapeutic agents like paclitaxel and doxorubicin in hypoxic tumors through polymeric nanodrug delivery systems, primarily by augmenting drug solubility, improving targeting, regulating drug release, and mitigating tumor resistance. Traditional cytotoxic agents like paclitaxel and doxorubicin exhibit inadequate aqueous solubility and therapeutic specificity. Polymeric nanodrugs can enhance drug solubility and ensure the optimal bioavailability of drugs in vivo. Incorporating these drugs into polymer nanoparticles not only improves drug stability but also extends circulation time in the bloodstream through PEGylation and other methods, thereby reducing immune system clearance and facilitating more efficient targeted delivery. The targeted recognition and uptake of tumor cells can be accomplished through the surface modification of antibodies, ligands, or small molecules. Polymeric nanomedicines exploit the characteristics of the tumor microenvironment, including hypoxia and acidity, to facilitate targeted drug release at the tumor site via pH-responsive or enzyme-responsive mechanisms.

**Figure 5:**
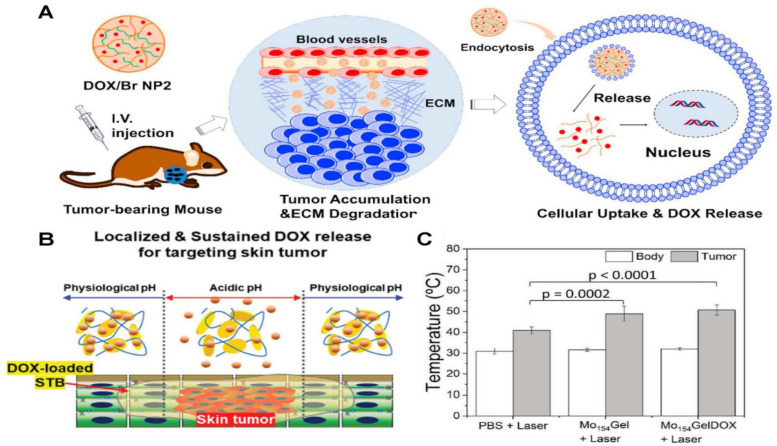
(**A**) The mechanism of nanoparticles (Br NP2) for enhancing DOX delivery by the degradation of tumor ECM [33], Copyright 2020, Elsevier; (**B**) schematic displaying pH-responsive DOX delivery using STBs for localized melanoma treatment [34], Copyright 2022, The Royal Society of Chemistry; (**C**) body and tumor temperature of the mice injected with PBS, Mo154Gel, and Mo154GelDOX upon 10 min of NIR irradiation [35], Copyright 2021, Wiley.

For example, Xin Wang et al. developed a novel pH-responsive nanocarrier (Figure 5A) by polymerizing bromelain (pineapple protease) with a neighboring ester group crosslinker [33]. The carrier was able to enhance drug penetration into tumor tissues by degrading the extracellular matrix in the tumor’s acidic environment. When the tumor is under acidic conditions, the nanocarrier degrades and releases free bromelain, which facilitates the penetration of adriamycin-containing nanoparticles into solid tumors. The nanoparticles exhibited a higher DOX release rate than non-sensitive nanoparticles, achieving 86% release within 120 h at pH 5.5. In vivo experiments demonstrated that pH-sensitive nanoparticles were degraded under weakly acidic conditions, and the released bromelain (Br) further facilitated nanoparticle penetration into the tumor parenchyma through ECM hydrolysis. Moreover, Br synergizes with DOX to impede tumor cell proliferation and elicit enhanced anti-tumor effects. Ultimately, these nanoparticles suppressed tumor growth by 62.5%, demonstrating the potential of bromelain-based stimuli-responsive nanomaterials as drug carriers for hypoxic tumor treatment.

The article by Lee, J. et al. in the journal *Nanoscale* focuses on a pH-responsive doxorubicin (Dox) drug delivery system specifically for local melanoma treatment (Figure 5B) [34]. Injectable shear-thinning biomaterials (STBs) have garnered significant interest due to their capacity to effectively and precisely administer cells, along with a diverse array of molecules, including growth factors and pharmaceuticals. Researchers have designed and synthesized an injectable STB with pH-responsive properties, which is capable of undergoing structural changes in acidic tumor microenvironments (low pH) to induce rapid drug release. As tumor tissues are usually weakly acidic, the system precisely releases doxorubicin at the tumor site, increasing the drug concentration and thereby effectively inhibiting the growth of melanoma cells. Studies using in vitro/in vivo trials and biocompatibility testing have revealed that this delivery system shows good biocompatibility and high efficacy in the local treatment of melanoma. It not only increases the anti-tumor effect but also reduces the negative side effects on normal tissues during chemotherapy, thereby offering a more focused and effective therapeutic approach for the treatment of hypoxic tumors.

#### 2.4.2. Photothermal, Photodynamic and Optical Immunotherapy

Especially in the treatment of hypoxic tumors, polymeric nanomedicines have shown great synergistic effects in combined photodiagnosis and immunotherapy. While polymeric nanomedicines can efficiently improve therapeutic efficacy and provide notable synergistic effects in photodiagnostics and immunotherapeutic treatments through targeted delivery and environment-responsive design, the unique microenvironment of hypoxic tumors (hypoxia, acidity, etc.) renders conventional therapeutic approaches ineffective due to their limited efficacy.

For example, the combination of phototherapy and polymeric nanomedicines can effectively enhance the therapeutic effect. In photothermal therapy, nanomedicines can carry photosensitizers that can be activated by an external light source to produce a thermal effect that destroys tumor cells. In hypoxic tumors, the low oxygen environment often leads to poor results of conventional treatments; however, photothermal therapy can break through this barrier by local heating, facilitating the release of chemotherapeutic drugs, while the photothermal effect enhances the destruction and death of tumor cells. In addition, polymeric nanomedicines are able to release chemotherapeutic drugs such as paclitaxel locally in the tumor in conjunction with photothermal therapy to enhance the anti-tumor effect of the drugs. The article by Guedes, G. (Figure 5C) [35] focuses on the design of a dual-crosslinked dynamic hydrogel combining a molybdenum-clustered compound (Mo154) with pH and near-infrared (NIR) responsiveness for combined chemotherapy and photothermal therapy. The researchers engineered and synthesized a double-crosslinked dynamic hydrogel featuring a crosslinked architecture that can respond to pH fluctuations in the tumor microenvironment and NIR photostimulation for regulated drug release. The Mo154 clusters exhibit excellent NIR light absorption and generate heat via the photothermal effect, thereby augmenting the efficacy of photothermal therapy. The hydrogel exhibits a commendable drug-loading capacity, effectively encapsulating doxorubicin (DOX) and facilitating pH-stimulated release in the tumor’s mildly acidic microenvironment. Furthermore, Mo154 converts NIR light into thermal energy **, ** inducing localized temperature elevation that enhances drug accumulation and release at the tumor site. The system markedly suppresses tumor growth and enhances therapeutic efficacy through the synergistic interaction of chemotherapeutic drug administration and near-infrared light irradiation. The article assessed the biocompatibility and safety of the hydrogel, revealing that it exhibits favorable biocompatibility and no significant toxic side effects, thus rendering it appropriate for application as a drug delivery system in tumor therapy. This innovative dual-crosslinked system integrates pH and NIR responsiveness, offering a precise and effective approach for combined chemo-photothermal therapy while minimizing toxicity to healthy tissues.

In addition, the combination of immunotherapy and polymeric nanomedicines can enhance the recognition and clearance of tumors by the immune system. Polymeric nanomedicines can activate the body’s immune response through targeted delivery systems, allowing drugs such as paclitaxel or doxorubicin to accumulate in high concentrations at the tumor site, inducing tumor cell death and releasing tumor antigens. At the same time, nanomedicines can carry immune-enhancing factors (such as cytokines or immune checkpoint inhibitors) to improve the effect of immunotherapy and enhance the ability of the immune system to recognize and remove tumor cells. The synergistic effect of immune checkpoint inhibitors and chemotherapeutic agents not only overcomes tumor immune escape but also enhances the immune system’s sustained attack against tumors. Photodynamic therapy (PDT) and immunotherapy combinations have shown promising results in enhancing cancer treatment outcomes. For instance, a study by Guedes et al. [35] demonstrated that a double crosslinked dynamic hydrogel combining a molybdenum—clustered compound (Mo154) with pH and near-infrared (NIR) responsiveness could effectively deliver doxorubicin and enhance photothermal therapy. The hydrogel exhibited commendable drug-loading capacity and facilitated drug release via pH stimulation in the tumor microenvironment. Moreover, the photothermal effect generated by Mo154 under NIR irradiation augmented the efficacy of the therapy by increasing localized temperature and promoting drug accumulation at the tumor site. This synergistic approach significantly suppressed tumor growth and enhanced therapeutic efficacy. Another example is the work by Zhang et al. [36], which presented a polymeric microsphere drug delivery system capable of encapsulating both immune checkpoint inhibitors and chemotherapeutic agents. The system was designed to enhance the therapeutic efficacy of glioblastoma by combining immunotherapy and chemotherapy. The results showed that the drug delivery system significantly enhanced drug accumulation at the tumor site, mitigated drug toxicity to normal tissues, and improved the anti-tumor immune response. These studies highlight the potential of combining photodynamic therapy and immunotherapy to overcome the challenges posed by hypoxic tumors and enhance treatment efficacy.

#### 2.4.3. Cytotoxicity Achieved by Other Means

The integration of polymeric nanomedicines with nucleic acid therapeutics, metal ions, and inorganic salts has shown considerable synergistic effects in the management of hypoxic tumors. Polymeric nanomedicines serve as carriers for nucleic acid therapeutics (e.g., siRNA or miRNA), significantly enhancing their stability and delivery efficacy. The hypoxic environment-responsive design enables the nanomedicine to precisely release nucleic acid drugs within the tumor microenvironment, suppress the expression of drug-resistant genes, and restore the responsiveness of tumor cells to chemotherapy, consequently enhancing the effectiveness of the treatment.

In addition, the combination of polymeric nanomedicines with metal ions (e.g., copper ions or iron ions, etc.) can enhance tumor cell death through mechanisms such as promoting oxidative stress or inducing ferroptosis. The nanomedicine carriers are able to release metal ions in a targeted manner in the tumor region, increasing the local oxidative stress and further improving the tumor response to chemotherapeutic agents. Simultaneously, metal ions can augment the efficacy of phototherapy and radiotherapy, creating synergistic treatments while reducing side effects.

The amalgamation of inorganic salts (e.g., calcium salts and phosphates) can modulate the pH or redox state of the tumor microenvironment, thereby enhancing tumor cell apoptosis. The binding of calcium ions and phosphates can enhance calcium signaling in tumor cells, leading to cell death. Polymeric nanomedicines improve drug release and ameliorate the tumor microenvironment by associating with these inorganic salts, thereby aiding in the mitigation of tumor immune evasion and drug resistance. Polymeric nanomedicines, in conjunction with therapeutic strategies such as nucleic acids, metal ions, and inorganic salts, offer a precise and effective multi-therapeutic approach for hypoxic tumors, anticipated to propel advancements in cancer treatment.

## 3. Typical Case Studies of Stimuli-Responsive Polymeric Nanomedicines in Oxygen-Depleted Tumor Therapy

### 3.1. Major Research Advances and Results for Hypoxic Tumors

Hypoxic tumors are prevalent features of the tumor microenvironment, wherein tumor cells adapt to the hypoxic conditions via metabolic pathways, subsequently influencing their growth, invasiveness, and therapeutic response. The difficulties posed by hypoxic tumors to treatment are primarily evident in the following aspects. The hypoxic environment facilitates tumor angiogenesis. Neoplastic cells promote neovascularization in hypoxic conditions by enhancing signaling pathways, including VEGF (vascular endothelial growth factor). Nonetheless, these newly formed blood vessels are frequently abnormally structured, leading to compromised blood flow and hindering the effective delivery of oxygen and pharmaceuticals to the tumor’s central region. This phenomenon diminishes the efficacy of pharmaceuticals, particularly chemotherapeutic agents, and prompts tumor cells to adapt to the hypoxic milieu, thereby intensifying tumor drug resistance. Furthermore, the hypoxic environment can facilitate genetic mutations and malignant transformations in tumor cells, thereby enhancing their invasiveness and metastatic potential. Consequently, therapeutic approaches aimed at the hypoxic tumor microenvironment have emerged as a significant focus in contemporary cancer research.

Recent years have witnessed substantial advancements in therapeutic approaches for hypoxic tumors. A range of intelligent nanodrug delivery systems has been developed utilizing the characteristics of the hypoxic environment. For instance, nanomedicine delivery systems developed from polymers or materials that degrade under hypoxic conditions can improve drug accumulation and release in response to hypoxia within the tumor or microenvironment. These drug delivery systems can proficiently address the challenges of drug administration posed by the inadequate vascular architecture of tumors and enhance the therapeutic efficacy of pharmaceuticals against tumors. Moreover, specific hypoxia-responsive pharmaceuticals and molecules, such as the hypoxia-inducible factor (HIF-1α) pathway, have emerged as novel targets. Regulating the HIF-1α pathway can impede the survival and proliferation of tumor cells and decelerate tumor progression. Additionally, combination therapy employs a synergistic approach by integrating immunotherapy and targeted therapy with chemotherapy and radiotherapy to augment the efficacy of tumor treatment. The integration of immune checkpoint inhibitors with conventional chemotherapeutic agents can significantly modulate the tumor immune microenvironment, thereby improving therapeutic efficacy.

Alongside advancements in drug delivery systems, novel therapeutic approaches like photothermal therapy and magnetic hyperthermia for hypoxic tumors are progressively achieving breakthroughs. For instance, employing nanomaterials (e.g., gold nanoparticles and titanium dioxide) as carriers for photothermal therapy exploits the elevated temperature response of tumor tissues to effectively eradicate tumor cells. The hypoxic environment allows these materials to attain an effective thermal response in the tumor microenvironment by improving the absorption of near-infrared light, subsequently leading to the destruction of tumor cell structures. Magnetic nanoparticles were incorporated into the treatment to augment drug accumulation in the tumor area and improve drug release efficacy via external magnetic field control. Furthermore, to target the metabolic pathways of tumor cells in an anoxic environment, metabolic inhibitors specifically designed for tumor cells were developed to impede their adaptation to hypoxia and improve treatment efficacy. These therapeutic modalities not only increase the sensitivity of tumors to pharmacological agents but also offer diverse clinical intervention options, signifying a novel phase in the research of hypoxic tumors.

### 3.2. Stimuli-Responsive Polymeric Nanomedicines with Potential for Clinical Translation

In recent years, nanotechnology has achieved remarkable advancements in drug delivery, particularly in overcoming the blood–brain barrier (BBB). Due to their distinct physicochemical attributes, which encompass a diminutive size, an elevated surface area–volume ratio, and adjustable surface properties, nanoparticles possess the capability to successfully traverse the blood–brain barrier (BBB). This ability facilitates improved drug distribution and increases therapeutic effectiveness within cerebral tissues. For instance, ferritin-based nanovehicles have been employed for brain tumor therapy, significantly improving treatment outcomes through RNA interference (RNAi) technology [80].

Despite the remarkable progress of nanoparticles in brain tumor therapy, their applications in treating other tumor types, particularly oral cancers, are equally significant. Oral cancer treatment faces multifaceted challenges, including local invasiveness, high recurrence rates, and resistance to conventional therapies. Nanoparticles can enhance therapeutic outcomes for oral tumors through the following mechanisms: (1) Enhanced Drug Delivery—nanoparticles improve drug permeability and retention within tumor tissues, thereby increasing therapeutic efficacy; (2) Targeted Therapy—engineered to selectively target cancer cells, nanoparticles minimize collateral damage to healthy tissues; (3) Overcoming Drug Resistance—nanoparticles circumvent tumor cell resistance mechanisms by delivering therapeutics directly to intracellular compartments via tailored pathways. These strategies highlight the transformative potential of nanoparticles in oral cancer treatment, offering promising avenues to amplify therapeutic effectiveness while reducing systemic toxicity.

#### 3.2.1. Drug Release Based on Hypoxic Activation

Polymer-based hypoxia-activated nanoparticle drug delivery systems activated under hypoxia (low oxygen conditions) are an innovative approach for targeted therapy, especially for cancer treatment. Tumors typically have areas of hypoxia due to abnormal blood vessel formation and high metabolic demands. These areas of low oxygen tension can be utilized to trigger the release of drugs from polymer nanoparticles, thereby increasing the specificity and effectiveness of treatment [37].

The fundamental principle of hypoxia-activated polymer nanoparticles is based on tumor hypoxia, which occurs due to inadequate oxygen supply resulting from abnormalities in the tumor’s vascular architecture. This hypoxic environment results in tumor degradation, metastasis, and resistance to therapies such as chemotherapy and radiotherapy. Hypoxia influences the expression of numerous proteins involved in the cellular stress response, notably hypoxia-inducible factor 1 (HIF-1), which governs gene regulation and facilitates angiogenesis, survival, and adaptation in hypoxic environments [81].

**Figure 6:**
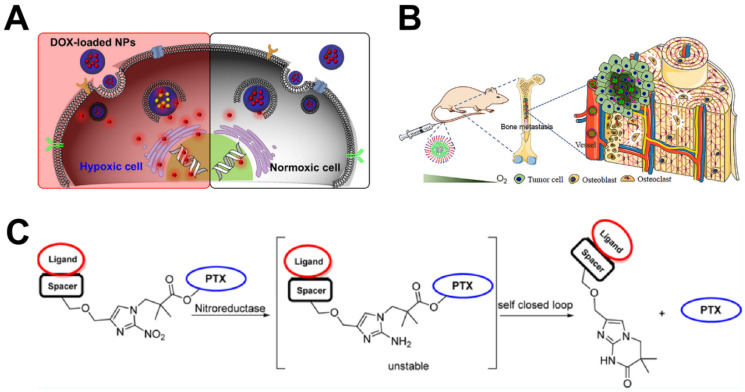
(**A**) Schematic diagram of the formation of HR-NPs and the in vivo tumor targeting pathway [38], Copyright 2013 Elsevier; (**B**) functionalized hypoxia-responsive polymeric micelles for targeted therapy of bone metastatic prostate cancer [39], Copyright 2021 Elsevier; (**C**) a novel bioinduced linker based on 2-nitroimidazole. -nitroimidazole is used for three paclitaxel (PTX) prodrugs, the 2-nitroimidazole linker can accelerate the release of prodrugs under hypoxic conditions [40], Copyright 2018 American Chemical Society.

Researchers created polymer nanoparticles that exploit hypoxic environments by releasing drugs in response to hypoxic microenvironments. Thambi et al. developed innovative hypoxia-responsive polymer nanoparticles (HR-NPs) for targeted drug delivery to tumors. HR-NPs selectively release doxorubicin (DOX) under hypoxia, with a 12 h DOX release rate of 49% under normoxia versus complete release under hypoxia. In vitro, HR-NPs showed low cytotoxicity toward SCC7 cells, whereas DOX-loaded HR-NPs exhibited significantly higher cytotoxicity under hypoxia, validating their therapeutic potential (Figure 6A) [38].

Oxygen-deficient polymer nanoparticles are achieved by incorporating hypoxia-sensitive linkers or portions into the nanoparticle structure. These linkers are stable under normoxic conditions but break or undergo conformational changes under hypoxic conditions, triggering the controlled release of the encapsulated drug. Some researchers have aimed to effectively treat bone metastatic prostate cancer by synthesizing a novel hypoxia-responsive polymeric microencapsulation system. Using a copolymer based on poly (vinyl alcohol) and poly(lysine), the researchers constructed this microcapsule by incorporating alendronate as the bone-targeting portion and azobenzene as the hypoxia-responsive connector. The system was highly sensitive to hypoxic conditions. In in vivo experiments, the microcapsules rapidly released the drug in a hypoxic environment to achieve an effective therapeutic dose, resulting in the inhibition of tumor growth, alleviation of bone pain, and prolongation of survival time in mice (Figure 6B) [39].

Hypoxia-sensitive linkers include nitroimidazole-based linkers, disulfide bonds, and azobenzene linkers, which undergo reduction or structural changes under hypoxic conditions, leading to drug release. Zhang et al. designed and synthesized a novel 2-nitroimidazole-based bioreductive linker for a ligand-targeted paclitaxel (PTX) prodrug system. The drug release mechanism was first verified by chemical reduction and nitroreductase assays, which demonstrated that in anoxic environments, 2-nitroimidazole can be reduced to 2-aminoimidazole and form a six-membered ring, thereby releasing the active drug PTX. The researchers combined the linker with known ligands (e.g., glucose and acetazolamide) to synthesize the Glu-PTX and AZO-PTX pre-drugs, and assessed their stability under physiological conditions and drug release ability under hypoxic conditions (Figure 6C) [40].

**Figure 7:**
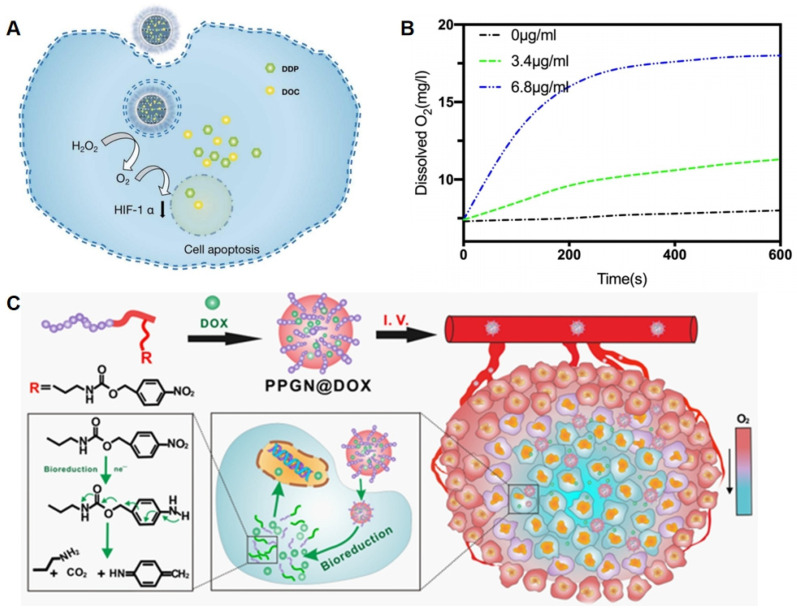
(**A**) Hollow mesoporous manganese dioxide (H-MnO_2_) nanoshells loaded with chemotherapeutic drugs docetaxel and cisplatin (TP), forming H-MnO_2_-PEG/TP nanoshells [41], Copyright 2021 BMC; (**B**) changes in O_2_ concentration in hydrogen peroxide solution (100 μM) [41], Copyright 2021 BMC; (**C**) a novel nanoparticle, this polymer is synthesized by coupling the hydrophobic small molecule 4-nitrobenzyl (3-azidopropyl) carbamate to the side chain of mPEG-PPLG copolymer [42], Copyright 2020 American Chemical Society.

The polymer matrix of polymer nanoparticles is typically biocompatible and biodegradable, enabling sustained drug release at the target site while minimizing damage to healthy tissue. Encapsulating the drug in hollow mesoporous MnO_2_ allows for controlled release upon reaching the hypoxic, acidic microenvironment of the tumor. This design resulted in enhanced precision of drug release control (Figure 7A) [41]. To validate the mechanism, researchers tested the catalytic ability of hollow mesoporous MnO_2_ to decompose hydrogen peroxide. An oxygen sensor was employed to quantify the amount of oxygen that was released into the solution following the introduction of hydrogen peroxide (100 μM) at varying concentrations of H-MnO_2_-PEG nanoshells. In the absence of H-MnO_2_-PEG nanoshells, the concentration of dissolved O_2_ in the hydrogen peroxide solution remained consistently low and stable. H-MnO_2_-PEG nanoshells can rapidly trigger the rapid generation of O_2_ from hydrogen peroxide in a concentration-dependent manner (Figure 7B) [41].

Other researchers have developed a novel nanoparticle, PPGN@DOX, which can selectively release the encapsulated chemotherapeutic agent doxorubicin (DOX) in hypoxic conditions for breast cancer treatment. PPGN@DOX demonstrated superior drug release under hypoxia and was effectively internalized by 4T1 breast cancer cells via endocytosis, enabling DOX delivery to the nucleus.PPGN@DOX exhibited hypoxia-sensitive drug release and significant anticancer efficacy in hypoxic environments, thereby reducing DOX systemic toxicity (Figure 7C) [42].

The benefits of hypoxia-activated polymer nanoparticles include selective delivery, circumvention of drug resistance, and reduced systemic toxicity. These systems facilitate precise drug delivery in hypoxic tumor areas, thus minimizing off-target effects and enhancing therapeutic efficacy. Tumors frequently exhibit resistance to standard treatments, including chemotherapy and radiotherapy, particularly in hypoxic areas. Hypoxia-activated nanoparticles can surmount this resistance by selectively administering therapeutic agents to regions that are least responsive to treatment. Since drug release is confined to hypoxic tumor tissues, healthy tissues avoid exposure to high drug concentrations, thereby mitigating systemic toxicity and side effects [43].

The potential for combination therapy is also an important aspect of hypoxia-activated polymer nanoparticles. The release of drugs sensitive to hypoxic conditions can be integrated with additional treatment strategies, including photodynamic therapy, immunotherapy, or radiotherapy [44,45].

Notwithstanding the promising notion of hypoxia-sensitive drug release, developing stable and efficient hypoxia-sensitive linkers that can be reliably cleaved under physiological conditions continues to pose a challenge. Investigations are ongoing to enhance the stability and specificity of these linkers. Tumors exhibit significant heterogeneity, with varying levels of hypoxia across different regions. This variability may influence the efficacy of drug release and therapeutic effectiveness. To resolve this issue, future designs may integrate multiple stimuli (e.g., pH, temperature, and hypoxia) to facilitate drug release across various tumor regions. Despite promising outcomes from in vitro and animal studies, additional research on safety, scalability, and regulatory approval is required to implement hypoxia-activated polymer nanoparticles in clinical practice.

#### 3.2.2. Therapeutic Mechanisms Utilizing Hypoxia-Inducible Enzymes

Polymer nanoparticles that utilize hypoxia-inducible enzymes for drug delivery are an advanced therapeutic strategy for the treatment of cancer and other hypoxic diseases. The principle behind this approach is to utilize the unique enzymatic activity triggered by hypoxic conditions to facilitate targeted drug release or activation of therapeutic agents at the disease site. Hypoxia-inducible enzymes are typically upregulated in the tumor microenvironment (TME), making them ideal targets for drug delivery systems that exploit this mechanism [37,82].

In hypoxic tumor microenvironments (TMEs), tumors typically have regions of hypoxia due to rapid growth and poor vascularization. These hypoxic regions are associated with increased expression of hypoxia-inducible factor (HIF), which activates a range of enzymes and proteins associated with tumor progression, angiogenesis, and metastasis. Hypoxia-inducible enzymes, such as hypoxia-inducible factor-1 (HIF-1), matrix metalloproteinases (MMPs), carboxylesterases (CESs), and cytochrome P450 enzymes (CYPs) [83], are activated by tumors in response to hypoxic environments, and they can be therapeutically targeted.

Polymeric nanoparticles can be designed to utilize these hypoxia-induced enzyme activities to achieve controlled and localized drug release. This is often achieved by incorporating into the nanoparticle structure enzyme-sensitive linkers or prodrugs into the nanoparticle structure, which remain stable under normal conditions but release drugs via enzyme-mediated cleavage in hypoxic regions. For example, peptide linkers can be cleaved by enzymes such as MMPs or other hypoxia-associated proteases to release encapsulated drugs in hypoxic tumors. In the prodrug design, inactive drugs are conjugated to polymeric nanoparticles and activated by hypoxia-induced enzymes [84].

Advantages of enzyme-mediated drug delivery using hypoxia-inducible enzymes include targeted and controlled release, overcoming drug resistance, minimizing systemic toxicity, and synergistic effects with other therapies [46].

The primary benefit of this strategy is the ability to target therapeutic agents to hypoxic tumor regions, which are generally the most aggressive and resistant to standard treatments. By shielding healthy tissues from drug exposure, treatment efficacy can be enhanced with minimized side effects. This strategy can be integrated with other treatments, such as radiotherapy or photodynamic therapy, to enhance the therapeutic effect.

Despite the preclinical promise of enzyme-activated polymer nanoparticles, further optimization is required for clinical translation. Key challenges include the following: (1) ensuring nanoparticle activation is exclusively triggered by tumor-specific enzymes; (2) addressing tumor heterogeneity; (3) guaranteeing nanoparticle stability, biocompatibility, and scalable production; (4) navigating regulatory challenges.

### 3.3. Typical Study Case Studies

#### 3.3.1. Key Research Advances and Results for Hypoxic Tumors

The development of polymer nanoparticle-based drug delivery systems has become a viable approach for cancer treatment considering the special properties of the tumor microenvironment (TME). Often resistant to conventional chemotherapy and radiotherapy, these hypoxic tumors are characterized by vascular dysplasia and an adaptive response of cancer cells to hypoxia. The targeting and controlled drug release made possible by polymeric nanoparticles show promise for overcoming these obstacles. Notably, the broad applicability of nanotechnology extends beyond the treatment of hypoxic tumors to demonstrate significant potential in managing other hypoxia-related diseases. By engineering specialized nanoparticle delivery systems, drug delivery efficiency for stroke treatment can be significantly enhanced. These systems can be optimized to adapt to blood–brain barrier (BBB) alterations, thereby improving drug penetration capacity and therapeutic efficacy [85].

**Figure 8:**
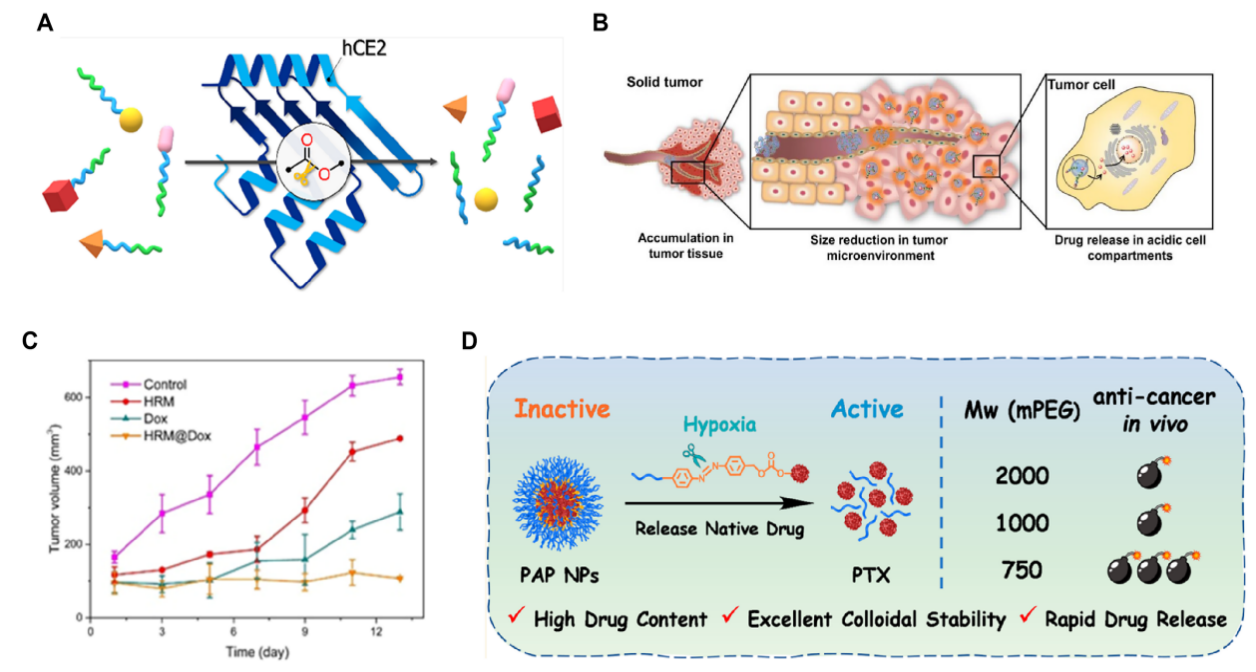
(**A**) Drug release via polymer–drug conjugates bound to human carboxylesterase 2 for targeted drug activation [47], Copyright 2023 American Chemical Society; (**B**) schematic diagram of the multi-stage drug delivery mechanism of pNG-DOX [86], Copyright 2020 Ivyspring International Publisher; (**C**) changes in tumor volume of mice during the treatment with HRM NPs [48], Copyright 2024 American Chemical Society; (**D**) schematic diagram of the self-assembly of PEGylated PTX prodrug, tumor hypoxia-specific drug release, and anti-tumor activity [87], Copyright 2022 American Chemical Society.

In polymer nanoparticle studies targeting hypoxic tumors, one approach to improve the selective delivery of drugs to hypoxic tumors is the use of hypoxia-inducible enzymes such as carboxylesterases (CES). These enzymes are more active in hypoxic TME and can control the release of prodrugs encapsulated in polymer nanoparticles. For example, carboxylesterase is highly expressed in certain cancer cells, especially in the hypoxic regions of the tumor, and researchers have developed polymer nanoparticle systems for encapsulating pre-drugs that are activated by carboxylesterase, leading to the selective release of the active drug in hypoxic regions (Figure 8A) [47]. In addition, hypoxic tumor regions usually overexpress matrix metalloproteinases (MMPs), and a novel multi-stage drug delivery system based on matrix metalloproteinase MMP-sensitive peptide-crosslinked nanogels (pNGs) has been developed with the aim of improving the delivery of anticancer drugs in three-dimensional tumor models. It was shown that such nanogels can be degraded by MMPs in the tumor microenvironment, leading to a size reduction in the particles and facilitating their penetration and distribution in tumor tissue. Experimental results showed that the size of the pNGs could be controlled by adjusting the crosslinking density and functionalization degree. MMP-mediated degradation not only enhanced their penetration into dense tumor tissues but also improved (e.g., doxorubicin) release efficiency (Figure 8D) [86].

Another significant advancement lies in the development of hypoxia-responsive drug delivery systems. These systems are specifically engineered to deliver therapeutic agents in hypoxic tumor regions via hypoxia-sensitive linkers or prodrugs that activate in low-oxygen conditions. For example, nitroimidazole-based linkers are stable under normal oxygen conditions but undergo a reduction in hypoxic environments, thereby activating a drug release mechanism that allows the nanoparticles to release the drug only when they reach the hypoxic tumor region. Some researchers have accelerated drug release by synthesizing hypoxia-responsive micelle nanoparticles (HRM NPs), which converted nitroimidazole to its hydrophilic form in hypoxic and reduced tumor microenvironment, leading to the disruption of micellar structure, which resulted in a cumulative release rate of 74.4% of DOX within 24 h. HRM@DOX nanoparticles have shown significant anti-tumor effects in vitro and in vivo. Under hypoxic conditions, the relative cell viability of HRM@DOX NPs was significantly lower than that of other controls, and the most significant inhibition of tumor volume was observed in the mouse model, with a tumor volume of only 106.9 mm^3^. These results indicate that HRM@DOX NPs enhanced anti-tumor activity by modulating the intracellular redox state, suggesting that they may be an effective new strategy in clinical cancer therapy (Figure 8B) [48]. Additionally, some researchers have developed a novel PTX prodrug nanoparticle (PAP NP) with good water solubility and biocompatibility to achieve selective drug release in the tumor hypoxic microenvironment. By introducing a cleavable coupling agent (azobenzene), PAP NPs were able to release paclitaxel more efficiently in tumor cells, thereby enhancing the anti-tumor effect. It was found that the cytotoxicity of PAP NPs against cancer cells under hypoxic conditions exhibited a markedly greater value compared to traditional PTX preparations, and the anti-tumor effect in mice was superior to that of the control group (Figure 8C) [87].

To enhance targeting specificity and augment drug release efficiency, researchers developed dual-responsive polymer nanoparticles. These nanoparticles are responsive to hypoxia and other tumor-specific factors, such as pH or temperature, undergoing structural/phase transitions under hypoxic or heated conditions to achieve spatiotemporally controlled drug release within tumors [88].

In addition, polymer nanoparticles are often designed to take advantage of the enhanced permeability and retention (EPR) effect—a phenomenon that refers to the selective accumulation of nanoparticles within tumor tissues, which can be attributed to the presence of leaky blood vessels and compromised lymphatic drainage, both of which are characteristic characteristics of tumors. Researchers have worked to improve the size, surface charge, and surface modifications of polymer nanoparticles to enhance their tumor-targeting efficiency, particularly in hypoxic regions [49,89].

Ultimately, polymeric nanoparticles aimed at hypoxic tumors may be integrated with complementary therapies to enhance efficacy. Significant combinations encompass chemotherapy and photodynamic therapy (PDT), immunotherapy, and radiotherapy alongside nanoparticle-mediated drug delivery. These combination therapies can surmount hypoxia-induced chemotherapy resistance and boost the efficacy of photodynamic therapy and radiotherapy in hypoxic tumors.

Significant research findings encompass preclinical investigations and clinical applications. Numerous preclinical investigations have validated the efficacy of polymer nanoparticle-based drug delivery systems in targeting hypoxic tumors, with certain systems progressing to early-stage clinical trials that have yielded promising preliminary outcomes regarding safety and efficacy.

Despite the significant potential of polymeric nanoparticle systems aimed at hypoxic tumors, challenges and future directions persist, including tumor heterogeneity, scalability, and manufacturing concerns. Future research will likely concentrate on creating multi-target delivery systems capable of responding to diverse stimuli and facilitating more efficient drug release within tumors, while also tackling the challenges of large-scale production and regulatory approval. These advancements are anticipated to address the limitations of traditional therapies and enhance the clinical outcomes for patients with refractory hypoxic tumors.

#### 3.3.2. Polymeric Nanomedicines with Clinical Translational Potential

The clinical application of polymeric nanodrug delivery systems targeting hypoxic tumors is an expanding field in cancer treatment. Researchers have achieved notable advances in creating nanoparticles that can efficiently target hypoxic areas of tumors, where traditional therapies often fail. Leveraging the unique characteristics of the TME for drug release offers the potential to address tumor drug resistance and improve patient outcomes [90].

Hypoxia-responsive polymer nanoparticles represent one of the most promising systems of polymer nanoparticles, which are specifically designed to release drugs under hypoxic conditions. For example, polyurea microcells with nitroimidazole linkers are capable of breaking down and releasing encapsulated drugs under hypoxia, whereas polymer nanogels containing hypoxia-responsive hydrazone bonds break under hypoxic tension, triggering drug release [37]. These systems have demonstrated good stability, biocompatibility, and enhanced drug release in preclinical models and are currently being investigated for their clinical applications.

Polymer nanoparticles targeting hypoxic tumors are increasingly being combined with other therapeutic modalities to achieve synergistic effects. These combinations include chemotherapy, photodynamic therapy (PDT), and immunotherapy. For example, polymer nanoparticles can be designed to carry both chemotherapeutic agents and photosensitizers for PDT, or to deliver immune checkpoint inhibitors or cytokines specifically to hypoxic tumor regions [91].

Hypoxic microenvironments diminish the efficacy of radiation-induced DNA damage; thus, polymer nanoparticles can mitigate the resistance of hypoxic tumors to radiation therapy by improving tumor oxygenation or functioning as radiosensitizers. Oxygen-releasing nanoparticles have demonstrated potential in preclinical trials for enhancing the effectiveness of radiation therapy.

Despite encouraging outcomes in preclinical investigations, the clinical application of these polymeric nanoparticle systems encounters obstacles including tumor heterogeneity, targeting efficiency, scalability, and regulatory challenges. As these systems progress through clinical trials and undergo optimization for safety, efficacy, and scalability, they are anticipated to improve clinical outcomes for patients with therapy-resistant hypoxic tumors. Nonetheless, issues concerning tumor heterogeneity, systemic toxicity, and production scalability must be resolved to guarantee their effective implementation in clinical settings. Polymer nanoparticle-based tumor-targeting systems possess considerable potential for clinical application due to their ability to selectively release drugs in tumor regions that are often resistant to conventional therapies. Innovations in hypoxia-responsive linkers, enzyme-activated prodrugs, MMP-sensitive nanoparticles, and combination therapies promote the advancement of more efficient and targeted approaches in cancer treatment.

## 4. Prospects for Stimuli-Responsive Polymeric Nanomedicines in Oral Cancer Therapy

### 4.1. Special Needs for Oral Cancer Treatment

#### 4.1.1. Impact of a Hypoxic Environment in the Treatment of Oral Cancer

In the treatment of oral cancer, hypoxic conditions have a significant impact on treatment efficacy and tumor behavior. Firstly, hypoxia induces radiation resistance, as oxygen is essential for fixing radiation-induced DNA damage [92,93]. Hypoxic cells are insensitive to radiation, which reduces the efficacy of radiation therapy, especially in head and neck cancers where tumors near critical structures limit safe radiation dosing.

Secondly, hypoxic environments also make oral cancer cells more resistant to chemotherapeutic agents, leading to shifts in cellular metabolism and weakened responses to certain chemotherapeutic agents [94]. In addition, hypoxia promotes the activation of molecular pathways and increases tumor aggressiveness, which is associated with enhanced invasion and metastasis. The upregulation of factors such as HIF-1α upregulation stimulates the expression of the genes related to cell migration, invasion, and angiogenesis, elevating metastatic risk [95].

Hypoxia is also associated with the enrichment of cancer stem cells, which are the main cause of tumorigenesis, recurrence, and drug resistance. They exhibit enhanced survival in hypoxic conditions and demonstrate insensitivity to standard therapies, resulting in tumor persistence and potential recurrence following initial treatment [96,97]. At the same time, the hypoxic tumor microenvironment leads to immunosuppression, reducing the ability of immune cells to infiltrate the tumor, suppressing the function of immune cells, and secreting immunosuppressive factors, limiting the effectiveness of immunotherapy [98,99].

In addition, areas of hypoxia induce the formation of new blood vessels (angiogenesis), but these are often irregular and poorly structured, leading to further areas of hypoxia and nutrient deficiency. This aberrant angiogenesis impairs the delivery of oxygen and therapeutic agents, increasing the complexity of treatment [100,101]. Hypoxia induces genetic instability, rendering cells more vulnerable to mutations and chromosomal rearrangements, which results in tumor heterogeneity and multidrug resistance [102].

Finally, the heterogeneity of tumor hypoxia, both across different cancers and within a single tumor, poses significant challenges for drug delivery. Variations in oxygen levels can lead to inconsistent drug release and therapeutic effects, as the responsiveness of stimuli-responsive nanomedicines may vary depending on the specific hypoxic conditions [103]. This highlights the need for personalized approaches and the development of nanocarriers that can adapt to the diverse microenvironments within tumors [104].

To address these challenges, hypoxia-targeted therapies have been the focus of research, including hypoxia-activated prodrugs (HAPs), which are active under hypoxic conditions and selectively kill hypoxic tumor cells [105]. Another strategy involves targeting the aberrant vasculature in hypoxic tumors by inhibiting angiogenesis, thereby normalizing the tumor vasculature, enhancing oxygen delivery, and augmenting the efficacy of additional treatments. Combination therapies, including the integration of conventional treatments with hypoxia-targeted agents or immunomodulators, are currently under investigation in clinical trials to address the difficulties presented by hypoxia in oral cancer [106].

In short, the hypoxic milieu of oral cancer enhances the tumor’s resistance to standard therapies, increases its aggressiveness, and augments its metastatic potential while enabling it to evade immune surveillance. Implementing targeted therapies or combination treatment strategies is expected to enhance the prognosis of patients with oral cancer.

#### 4.1.2. Importance of Local Delivery and Targeted Therapeutic Strategies

Local delivery and targeted therapeutic approaches are gaining significance in the management of oral cancer as they enhance treatment efficacy while reducing side effects. Local drug delivery refers to the administration of therapeutic agents directly to the tumor site, as opposed to depending on systemic circulation. This approach offers key advantages such as increased drug concentration, reduced systemic toxicity, controlled drug release, and treatment of inoperable tumors. For example, local delivery allows the drug to accumulate at the tumor site at a higher concentration, increasing its effectiveness, while minimizing the exposure of healthy tissue to the drug and reducing the potential for systemic side effects [107,108]. In addition, local delivery systems can be designed for sustained release of the drug, prolonging the exposure time of the drug within the tumor and improving the overall therapeutic effect [109,110].

Targeted therapies concentrate on particular molecular targets or signaling pathways that play a crucial role in tumor proliferation and maintenance, making them especially significant for oral cancer. These therapies enhance treatment accuracy, minimize adverse effects, and assist in overcoming drug resistance. For example, oral cancer cells often have specific molecular markers, such as EGFR (Epidermal Growth Factor Receptor) or human papillomavirus-associated markers, and targeted therapies can selectively target these markers to inhibit cancer cell growth while protecting normal cells [111,112]. In addition, targeted therapies can be used in combination with other therapies to improve overall efficacy [113].

The combination of localized drug delivery with targeted therapies allows for greater benefits such as increased drug accumulation, enabling precise treatment, and overcoming barriers. This combination ensures higher drug concentrations in the cancer cells while reducing systemic exposure, enabling highly specific therapies and minimizing damage to normal tissues [114].

EGFR-targeted therapies have proven particularly efficacious in HPV-related oral cancers, utilizing agents like cetuximab and erlotinib to obstruct EGFR signaling and impede tumor proliferation and metastasis. No literature was identified. Concurrently, nanoparticle-based delivery systems are being engineered to administer chemotherapeutic or targeted agents directly to the tumor site, regulating drug release and ensuring prolonged interaction with cancer cells while reducing side effects. The integration of immunotherapy and targeted delivery may enhance the capacity of immune cells to identify and eradicate cancer cells, especially in immune-evasive tumors.

The integration of localized delivery and specific therapeutic approaches is essential in the management of oral cancer. These approaches not only enhance the concentration of the drug at the tumor location while reducing systemic adverse effects, but they also guarantee that the therapy is specifically targeted towards the cancerous cells. The integration of these strategies offers oral cancer patients a more effective, precise, and individualized treatment regimen, which is anticipated to enhance treatment outcomes while minimizing side effects.

### 4.2. Potential Advantages of Stimuli-Responsive Polymeric Nanomedicines

#### 4.2.1. Enhancement of Drug Permeability and Absorption

**Figure 9:**
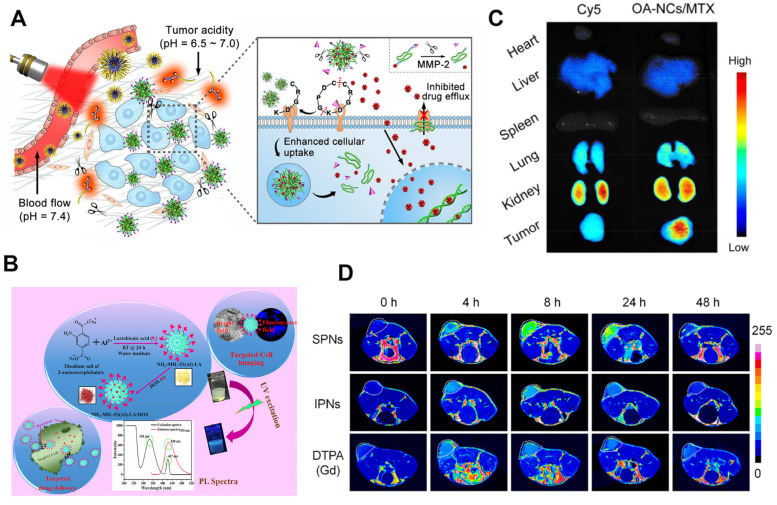
(**A**) Schematic illustration of tumor acidity-mediated activation of iPAPD nanoparticles with iRGD-enhanced tumor penetration and imaging-guided combination cancer therapy [122], Copyright 2017, American Chemical Society; (**B**) schematic diagram of in situ synthesized LA-targeted NMOFs for bioimaging and targeted drug delivery application [125], Copyright 2019, Elsevier; (**C**) fluorescent quantification of Cy5 in tumor and major healthy organs ex vivo 24 h post mPEG-OA administration [131], Copyright 2018, American Chemical Society; (**D**) in vivo MR images of EMT6 tumor-bearing mice after intravenous injection of SPNs, IPNs, and DTPA(Gd), respectively [132], Copyright 2020, American Chemical Society.

The use of stimuli-responsive polymer nanomedicines (SRPNs) has been shown to be an efficient means of enhancing the penetration and absorption of drugs, particularly in applications for the treatment of cancer, especially oral cancer [115,116], where they enhance drug penetration. Firstly, temperature- or pH-sensitive polymers can adjust their structure according to environmental changes, enhancing the ability of nanoparticles to cross biological barriers, such as the oral mucosal barrier, and releasing the drug in a controlled manner upon arrival at the target tumor site [117]. Secondly, the unique characteristics of the tumor microenvironment, such as acidic pH [118,119], allow stimuli-responsive polymer nanoparticles to release drug active ingredients in the tumor region, such as inducing the production of ROS [120], which increases the concentration of the drug in the tumor tissue and enhances the permeability and targeting [121]. Wang et al. described a novel tumor acid-triggered ligand presentation (ATLP) nanoparticle designed for cancer therapy. These ATLP nanoparticles consist of an acid-sensitive diblock copolymer that acts as a removable matrix, along with an iRGD-modified adriamycin polymer prodrug (iPDOX) that serves as an amphiphilic core. The nanoparticles preferentially accumulate at tumor sites due to the enhanced permeability and retention (EPR) effect, thereby enabling the acid-triggered nanoparticles to function effectively within the tumor microenvironment. Following this accumulation, the polymer matrix undergoes acid-induced dissociation in the tumor’s acidic environment (approximately pH 6.8), which exposes the iRGD ligand, thereby improving tumor penetration and the cellular uptake of the PDOX prodrug. This acid-mediated dissociation of the polymer matrix led to a 4.5-fold increase in the fluorescence signal, facilitating the in vivo monitoring of nanoparticle activation. Furthermore, upon exposure to near-infrared (NIR) laser irradiation, the activation of Ce6 significantly promoted the generation of reactive oxygen species (ROS), which in turn enhanced drug diffusion throughout the tumor mass and addressed acquired resistance by altering the gene expression profile of the tumor cells (Figure 9A) [122]. In addition, designing nanoparticles with specific size and surface charge characteristics enables them to swell in response to changes in pH or ionic strength, penetrating the extracellular matrix to penetrate deeper into the tumor [123,124].

SRPNs significantly enhance drug absorption. They prevent premature drug degradation, as numerous oral cancer medications are susceptible to decomposition in the gastrointestinal tract or systemic circulation. Nanoparticle-conjugated targeting ligands, such as the organic molecule lactobionic acid (LA), preserve the drug’s integrity until it reaches the site of action. Samui et al. reported that the LA-modified nanoscale metal–organic frameworks (NH2-MIL-53(Al) NMOFs) exhibited excellent stability and biocompatibility, effectively targeting desialylated glycoprotein receptors prevalent in human hepatocellular carcinoma cell lines, and demonstrated enhanced cytotoxicity against HepG2 cell lines. NH2-MIL-53(Al) NMOFs possess intrinsic fluorescent characteristics. The fluorescence intensity remains constant following LA conjugation, yielding effective therapeutic outcomes in cellular imaging and targeted drug delivery (Figure 9C) [125]. Once the nanoparticles reach the target tissue structure, they release the drug in response to specific stimuli in the microenvironment [126]. Controlled and triggered drug release is a major advantage [127], which enhances the bioavailability of the drug in the area of action and reduces systemic exposure [128,129,130]. For instance, acidic pH in the oral cavity or tumor area triggers the polymer matrix to break down, releasing the drug and prolonging its blood circulation, thereby increasing drug delivery to tumor tissues. For example, Tao et al. reported an amphiphilic mPEG-OA prodrug conjugate of oleic acid (OA) physically encapsulated with a model drug (methotrexate/MTX) and covalently linked to methoxy poly (ethylene glycol) (mPEG) via a stilbene linker, which self-assembles into pH-responsive micelles. The intensity of the free prodrug was significantly lower (*p* < 0.05) than that of the micellar nanocarriers from 3 h to 24 h after administration, which is thought to be a result of the extended blood circulation of the micellar nanocarriers. The micelles were stable at pH 7.4, and in the acidic endosomal/lysosomal microenvironment, the decomposition of stilbene induced micelle disintegration and the rapid release of OA and MTX, demonstrating significant tumor growth inhibition in vivo (Figure 9B) [131]. Meanwhile, the efficacy of drugs to cross the mucosal barrier can be enhanced by tuning nanoparticle properties, such as pH-responsive polymers adjusting their surface charge or swelling behavior depending on the acidic and alkaline environment of the oral mucosa.

SRPNs facilitate precise delivery and mitigate adverse effects. They can be engineered to release active compounds exclusively in reaction to specific environmental stimuli. For instance, Hao et al. created stimuli-responsive polymeric nanoparticles (SPNs) loaded with camptothecin (CPT) for deep tumor penetration. These targeted nanoparticles (SPNs) exhibit a sequential response to the acidic microenvironment and heightened reducing conditions present within the cytosol of tumors. Upon their arrival at the tumor location through the bloodstream, SPNs are capable of accumulating effectively and remaining at the acidic tumor site. This accumulation facilitates the dissociation into smaller, positively charged polypremedicine nanoparticles, which are essential for achieving substantial tumor penetration and improved cellular uptake. Ultimately, the parent drug, CPT, is released within the reducing environment of the cytoplasm, thereby facilitating the activation of chemotherapy. Notably, during these processes experienced by the nanocarriers in vivo, SPN exhibited progressively enhanced MR signals, which are promising for diagnosis and endogenous activation-mediated therapy (Figure 9D) [132].

Moreover, they can improve drug stability and regulate release. Numerous pharmaceuticals are susceptible to environmental degradation during transportation; SRPNs can stabilize these drugs—particularly poorly water-soluble agents or biologics—through protective encapsulation [133]. In addition, the nanoparticles can be designed to release the drug in a controlled manner during a specific period of time in response to a stimulus, maintaining a high concentration of the drug in the target area, improving absorption efficiency, extending the therapeutic window, reducing the need for frequent dosing, and enhancing patient compliance and reducing side effects.

Finally, SRPNs can enhance cellular uptake. A number of stimuli-responsive polymers, whose structures can be adapted to environmental changes, can enhance the interaction of nanoparticles with cell membranes [134]. For example, pH-sensitive nanoparticle cationization under acidic conditions enhances the interaction with negatively charged cell membranes and accelerates the endocytosis process. Conformational changes in the tumor region can also enhance the endocytosis activity of cancer cells, optimizing drug delivery to the cytoplasm or nucleus and enhancing the therapeutic effect. In conclusion, stimuli-responsive polymeric nanoparticles have many advantages in therapeutic areas such as oral cancer, with the potential to revolutionize treatment efficacy through enhanced targeting and controlled drug release.

#### 4.2.2. Reduction in Toxic Side Effects of Treatment on Normal Tissues

SRPNs have demonstrated significant advantages in reducing the toxic side effects of cancer treatments on normal tissues [135]. These nanoparticles are able to release drugs precisely in response to specific stimuli in the tumor microenvironment (e.g., pH, enzyme activity, redox status, or hypoxia levels), effectively minimizing off-target effects. By enabling tumor-specific drug release, SRPNs reduce the systemic exposure of healthy tissues to chemotherapeutic agents [136], thereby decreasing common side effects of chemotherapy and radiotherapy such as nausea, fatigue, and hepatorenal toxicity. This precision delivery system enhances therapeutic efficacy while sparing normal tissues [137,138].

SRPNs can reduce systemic toxicity via localized delivery and controlled release. Conventional therapies often result in high drug concentrations that can adversely affect healthy tissues; however, SRPNs can target the tumor site directly and release drugs gradually, maintaining a consistently effective therapeutic concentration [139]. This strategy prevents peak plasma concentrations from harming healthy tissues and reduces the need for frequent dosing, thereby reducing the associated toxicities [140,141].

In addition, SRPNs further reduce damage to healthy tissues by protecting drugs from premature degradation. For example, certain unstable chemotherapeutic drugs may degrade in the bloodstream or gastrointestinal tract, producing harmful by-products [142]. SRPNs can encapsulate these pharmaceuticals and safeguard their integrity, ensuring that the drug is released exclusively at the tumor site. Moreover, SRPNs can be fabricated using biocompatible and non-toxic materials [143], ensuring that the nanoparticle carriers themselves do not negatively affect normal tissues [144].

SRPNs demonstrate significant potential for organ-specific protection and immune regulation. In oral cancer, SRPNs can be engineered to permeate the oral mucosa without inducing local irritation, thereby markedly diminishing side effects such as oral ulcers resulting from chemotherapy and radiotherapy [143,145]. At the same time, SRPNs can avoid the activation of the immune system and reduce the risk of inflammation [146,147]. These features ensure that the function of normal tissues is not affected by the drugs, providing a safer option for cancer treatment [148].

In conclusion, stimuli-responsive polymeric nanoparticles offer an effective solution to reduce the toxicity of cancer therapy by enabling personalized and precise drug delivery [149,150]. They possess the capability to create targeted release systems tailored to the unique characteristics of a patient’s tumor. This approach minimizes drug exposure to surrounding healthy tissues, thereby enhancing the overall effectiveness of the treatment [151,152]. This innovative technology enhances anticancer effects while protecting normal tissues, positioning SRPNs as a safer and more effective treatment paradigm—particularly for oral cancer [153].

### 4.3. Innovative Technologies and Future Directions

#### 4.3.1. Multifunctional Combined Nanomedicine-Based Therapeutic Programs

SRPNs have shown great potential in the development of multifunctional combination therapies, providing innovative solutions for cancer treatment [154]. SRPNs can accurately deliver and regulate the release of drugs in reaction to particular stimuli within the tumor microenvironment, including pH, enzyme activity, or redox status. This characteristic provides them a considerable advantage in integrating diverse therapeutic modalities, including chemotherapy, immunotherapy, photodynamic therapy (PDT), and radiotherapy. SRPNs are engineered with multiple functions that improve therapeutic effectiveness while reducing the negative impacts of traditional therapies on healthy tissues.

The combination of chemotherapy and targeted therapy is one of the core strengths of SRPNs. These nanoparticles can be designed to release drugs in the acidic microenvironment of a tumor [155] while delivering targeted agents that synergistically act against specific receptors on the tumor surface [116]. For example, the combination of chemotherapy, which damages the DNA of tumor cells, and targeted therapies, which inhibit specific growth pathways in tumors, can significantly improve therapeutic efficacy and reduce the risk of drug resistance [156]. Furthermore, SRPNs facilitate precise delivery to designated areas of the tumor, thereby circumventing the systemic toxicity of chemotherapeutic agents to healthy tissues.

In the combination of immunotherapy and PDT, SRPNs further optimize the treatment paradigm. SRPNs can deliver both immune checkpoint inhibitors (e.g., PD-1 inhibitors) and photosensitizers [157] by stimulating the patient’s immune system to recognize and attack the cancer cells while at the same time using the ROS generated by the PDT to inflict a direct kill on the tumor cells [158,159]. The synergistic effect of the two not only enhances the efficacy of tumor therapy, but also effectively overcomes the limitations of PDT due to poor light permeability [160]. The targeted release of SRPNs in the tumor microenvironment markedly reduces systemic toxicities [161].

#### 4.3.2. Future Directions in the Development of Novel Stimulus Responsive Materials

**Figure 10:**
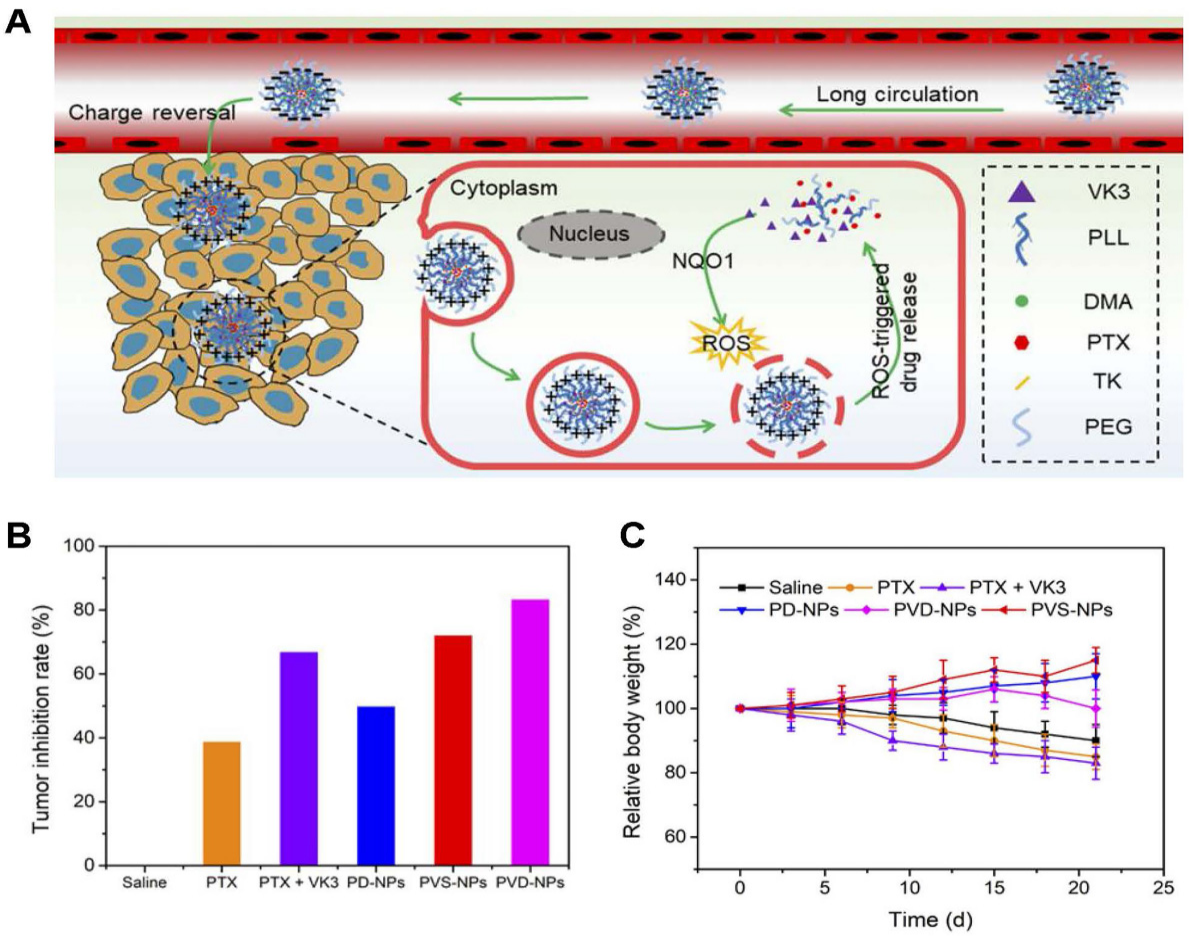
(**A**) The illustration of the charge-reversal and intracellular ROS generation of PVD-NPs in tumor tissue [163], Copyright 2020, DOVE Medical Press; DOVE MEDICAL PRESS LTD; Dove; (**B**) tumor inhibition rate of mice after treatment with different formulations [163], Copyright 2020, DOVE Medical Press; DOVE MEDICAL PRESS LTD; Dove; (**C**) mouse body weight after treatment with different formulations [163], Copyright 2020 DOVE Medical Press; DOVE Medical Press Ltd.; Dove.

In the future, the development of SRPNs will further focus on multi-stimuli-responsive systems and personalized design [149]. Multi-stimulus response systems enable precise drug release based on tumor dynamics, such as dual triggering mechanisms combining acidic pH and enzymatic activity [162]. Customized therapeutic protocols can be adapted to the patient’s tumor attributes to guarantee the optimal timing and dosage of drug administration, thereby improving efficacy and minimizing adverse effects. Xu et al. developed pH/ROS dual-responsive drug delivery platforms (PVD-NPs) featuring surface charge reversal and self-accelerating drug release by encapsulating VK3 within pH/ROS dual-responsive micellar nanoparticles. Following intravenous administration in mice, the PVD-NPs demonstrated prolonged blood circulation time and effectively retained a negative surface charge. The PVD-NPs’ reaction to the acidic tumor microenvironment resulted in a transition to a positive surface charge, thereby facilitating enhanced internalization by cancer cells in vivo. Ultimately, endogenous ROS can initiate the release of PTX and VK3, while the released VK3 stimulates ROS production. This self-reinforcing cycle enhanced tumor suppression and reduced systemic toxicity (Figure 10) [163]. However, despite the promising results in preclinical studies, the clinical translation of these nanomedicines faces significant challenges. One of the primary obstacles is the discrepancy between preclinical and clinical trial outcomes. Many nanomedicines that perform well in preclinical studies fail to meet the expected efficacy and safety profiles in clinical trials. This is often due to the complexity of the human body and the heterogeneity of tumors in patients. For instance, the multifunctional design of nanomedicines, while promising in preclinical studies, can lead to increased regulatory and translational complexity, higher costs, and difficulties in reproducibility [164]. A notable example of a nanomedicine that has successfully translated to clinical use is Vyxeos (CPX-351), a multilamellar liposome encapsulating cytarabine and daunorubicin in a 5:1 ratio. Vyxeos has shown improved overall survival in older patients with secondary acute myeloid leukemia [165]. However, other multifunctional nanomedicines, such as those designed for cancer stem cell ablation or theranostic applications, face significant challenges in clinical trials. These challenges include issues related to batch-to-batch consistency, high endotoxin content, and potential adverse effects such as cytokine storm, hypersensitivity, thrombosis, and immunotoxicity. Another challenge is the classification of multifunctional nanomedicines as medical devices, which poses additional regulatory hurdles [166]. These nanomedicines often require sophisticated analytical and toxicological approaches to validate their safety and efficacy, further complicating the clinical translation process [167]. Additionally, the high cost of nanomedicines compared to standard treatments can impose a significant financial burden on patients and insurers, especially when the efficacy in clinical trials is relatively low [168]. To address these challenges, future research should focus on simplifying the design of nanomedicines to enhance their reproducibility and reduce costs. Additionally, robust preclinical models that better mimic the human tumor microenvironment are needed to improve the predictability of clinical outcomes. Close collaboration with regulatory agencies and adherence to Good Manufacturing Practices (GMPs) will also be crucial for ensuring the successful translation of these nanomedicines to clinical use [169].

The tunability and flexibility of SRPNs not only make them ideal vehicles for combination therapy, but also open up new possibilities for the treatment of complex cancers [170]. Through deep integration with multiple therapeutic modalities, SRPNs are expected to become an important tool for future cancer treatment [171]. The development of SRPNs will also show great potential for intelligent, multifunctional, and personalized design. Future directions for SRPNs for the complex microenvironment of hypoxic tumors include the development of dynamic and smart materials that can respond to a variety of stimuli (e.g., pH, hypoxia, enzyme activity, and temperature), as well as “all-in-one” platforms combining diagnostic and therapeutic functionalities [172,173]. These materials can not only regulate drug release in real time to improve the precision of treatment, but also personalize the treatment by adapting to the tumor characteristics of different patients through multimodal design [174,175]. Furthermore, multifunctional synergistic therapeutic platforms will be a primary emphasis of research and development, including the amalgamation of oxygen-releasing agents and oxygen-depleting activating drugs within a single nanocarrier, as well as the integration of chemotherapy, radiotherapy, or immunotherapy, which can further augment therapeutic efficacy and overcome tumor resistance [176].

In order to achieve clinical translation, future focus will also need to be placed on the biocompatibility and degradability of SRPNs, optimizing their long-term toxicity assessment, as well as accelerating the design of nanomaterials and optimization of drug delivery strategies through artificial intelligence and big data technologies [143]. In addition, standardized manufacturing and international regulatory compliance are key to ensuring large-scale applications [177]. The practical application of SRPNs will become more feasible through the development of efficient manufacturing processes and the establishment of uniform quality control standards [178]. In this context, the stability of nanomaterials is also a crucial factor that needs to be addressed. Future research should focus on enhancing the stability of nanomaterials, particularly in terms of their long-term stability and safety in clinical applications. By optimizing the design and fabrication processes of nanomaterials, their stability and biocompatibility can be significantly improved, thereby enhancing their potential for oral tumor treatment. For example, introducing new stabilizers or surface modification techniques can enhance the stability of nanoparticles in the bloodstream and improve drug release efficiency, leading to better therapeutic outcomes and reduced side effects [179]. In conclusion, future SRPNs will provide more effective and safer therapeutic options for hypoxic tumors, promote the development of precision medicine, and offer brand new hope to patients with refractory cancers [180].

## 5. Existing Problems and Challenges

### 5.1. Bottlenecks in the Design Optimization of Stimuli-Responsive Nanomedicines—The Balance Between Drug Loading, Release Efficiency and Targeting

The design and optimization of SRPNs encounter numerous challenges in cancer therapy, particularly in achieving a balance among drug-loading capacity, release efficiency, and targeting specificity. The equilibrium among these three factors is essential to guarantee the safety and effectiveness of nanoparticles in clinical applications. In practice, these factors frequently interact, rendering the optimization of the design highly complex. While increasing drug loading can enhance therapeutic efficacy [181], it may lead to decreased stability or limited release control of the nanoparticles [182,183]. Consequently, the advancement of SRPNs that combine elevated drug-loading capacity with controlled release and high targeting specificity has emerged as a critical objective.

**Figure 11:**
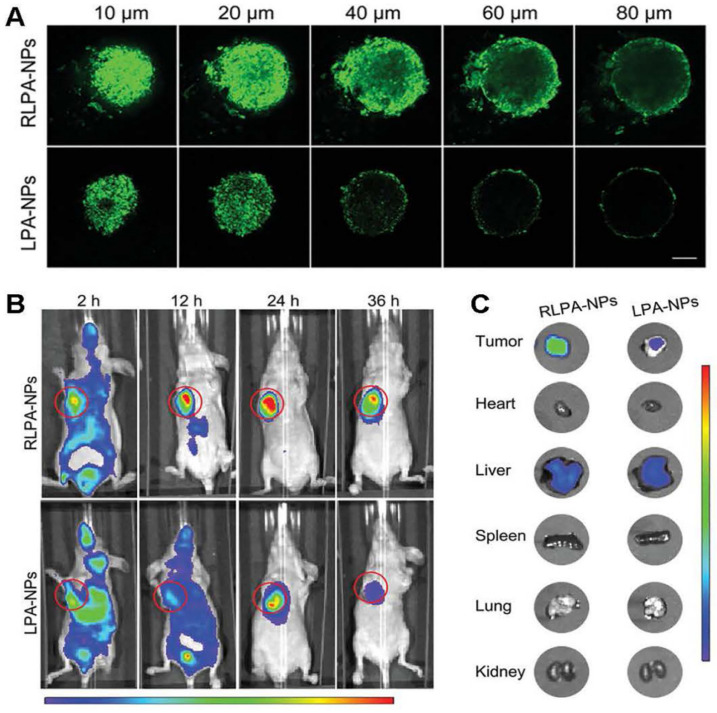
(**A**) In vitro tumor penetration of RLPA-NPs and LPA-NPs into 4T1 3D TSs after 8 h incubation. Green represents FITC. scale bar: 50 µm (reproduced from [185]). (**B**,**C**) In vivo (**B**) and ex vitro (**C**) images of 4T1 tumor-bearing mice following the systemic administration of Cy5.5-loaded RLPA-NPs and LPA-NPs for indicated times (reproduced from [185]).

The enhancement of drug release efficacy is a fundamental component of SRPN design. To attain precise release, researchers have investigated various trigger-responsive materials, including polymeric substances that respond to acidic pH, temperature, or enzymatic activity. These materials can undergo structural alterations in response to stimuli from the tumor microenvironment to release the drug [184]. Nevertheless, depending solely on a singular stimulation mechanism may prove inadequate to address the complexities of the tumor environment. Consequently, the incorporation of diverse stimulus–response mechanisms, such as the dual responsiveness to pH and temperature, can enhance the precision of drug release control. This ensures that the medication is delivered to the targeted site at the most effective time and dosage, which in turn increases therapeutic efficacy and reduces adverse effects. Li et al. have created a cascade-amplified drug release nanosystem responsive to both pH and reactive oxygen species (ROS), referred to as RLPA-NP. Upon intravenous administration, these nanoparticles accumulate in tumor tissues through active targeting mediated by iRGD and the enhanced permeability and retention (EPR) effect, subsequently penetrating further into the tumor mass (Figure 11) [185]. The RLPA-NPs are then taken up by cancer cells via receptor-mediated endocytosis. Within the acidic conditions of the lysosomal environment, OA-P (Asp-API) transitions to a hydrophilic state, facilitating the escape of RLPA-NPs from the endosomes through the “proton sponge effect”. Concurrently, RLPA micelles undergo disassembly, resulting in the release of Lap, which in turn permits the liberation of paclitaxel (PTX) precursors. Following this, the presence of endogenous ROS triggers the release of PTX from these precursor drugs. The liberated Lap is then catalyzed by NQO 1 to generate ROS, further amplifying and accelerating the release of PTX to induce cytotoxicity in tumor cells.

Target specificity presents a significant challenge. The heterogeneity of the TME, vascular permeability, and variability in tumor marker expression complicate the design of nanoparticles with high specificity [186]. Functional modification of the nanoparticle surface, such as the addition of antibodies, peptides, or other targeting ligands, can significantly improve selectivity for tumor tissue [187]. However, such highly specific designs may reduce the overall distribution range of the nanoparticles, resulting in less efficient drug delivery [188]. Integrating passive targeting (e.g., EPR effect) with active targeting strategies [189], combined with biocompatible coatings (e.g., polyethylene glycol) that extend circulatory persistence [190], enables optimal balancing between targeting precision and delivery efficacy.

Researchers are investigating diverse innovative strategies to optimize drug loading, release efficiency, and targeting. For example, hierarchically structured nanoparticles can carry high drug loading in the core region, while the outer layer is designed to release the drug in response to stimulation [191]. Furthermore, the hybridization of lipids, polymers, and inorganic materials can amalgamate the benefits of each material type to attain an organic integration of loading capacity, release efficiency, and target specificity [192]. Through advanced in vivo testing and computational modeling, the interaction of nanoparticles with the tumor microenvironment can be simulated to further optimize the design [193]. With the continuous advances in nanotechnology, materials science, and drug delivery strategies, SRPNs are expected to become safer and more efficient drug delivery tools, significantly improving the therapeutic efficacy of diseases such as cancer [194].

### 5.2. Limitations of Animal Models and Clinical Translation

#### 5.2.1. Inadequate Methods for Modeling and Assessing the Hypoxia Microenvironments

Accurately modeling and assessing hypoxia in the tumor microenvironment is a major challenge in cancer research. Hypoxia is a common feature of many solid tumors, including oral cancers, and plays an important role in tumor biological behavior, therapeutic response, and drug resistance. However, the existing animal models have significant limitations in reproducing the hypoxic microenvironment. For example, tumor vasculature in animal models is typically more regular than in human tumors [195], lacking the irregularity and chaotic blood flow evident in human tumors [196], which makes animal tumors less hypoxic than human tumors [197]. Furthermore, the accelerated tumor growth rates observed in animal models [198] often generate hypoxic regions that do not mirror the gradual hypoxic progression in human tumors, thereby compromising the faithful representation of hypoxia complexity [199].

Existing tools for assessing hypoxia also suffer from technical bottlenecks. Commonly used hypoxia markers, such as pimonidazole and EF5, while capable of labeling areas of hypoxia [200], often fail to reflect changes in dynamic oxygen levels within the tumor in real time [201]. In addition, imaging techniques such as PET and MRI, while providing a possible means of assessing hypoxia, have limited resolution and tissue penetration to capture micro-regional hypoxic features within the tumor [202]. The inadequacy of these tools makes it significantly difficult for scientists to study the dynamics of the hypoxic microenvironment and the effects of treatment on hypoxia, especially when tracking changes in hypoxic regions during treatment [203].

The assessment of dynamic hypoxia is even more of a challenge in current research. The level of hypoxia within a tumor is not static but fluctuates with tumor growth, metabolic changes, and therapeutic interventions [98]. For example, treatments such as radiotherapy may initially exacerbate hypoxia and may improve oxygenation in the long term [204]. Nonetheless, the current markers and imaging technologies are inadequate for the real-time monitoring of these changes, thereby constraining a thorough comprehension of treatment effects on the tumor microenvironment. During chemotherapy, radiotherapy, or immunotherapy, the dynamic response of the hypoxic microenvironment is frequently closely linked to efficacy; however, the existing assessment methods fail to accurately capture this intricate dynamic change [205].

To address these challenges, forthcoming research necessitates the creation of more sophisticated animal models and evaluation instruments that can more precisely simulate and monitor the hypoxic microenvironment in human tumors. This encompasses the creation of models that replicate the intricate vascular characteristics of human tumors, imaging methodologies that continuously assess oxygen levels in real time, and integrated strategies that amalgamate biomarkers with high-resolution imaging. Enhancing the methods for studying the hypoxic microenvironment can yield insights into the role of hypoxia in tumor progression and treatment, thereby establishing a more robust scientific foundation for optimizing therapeutic strategies.

#### 5.2.2. Challenges in the Transition from Laboratory Research to Clinical Application

The transition of SRNPs from laboratory research to clinical applications encounters numerous challenges, particularly in the domain of hypoxic tumor therapy. The intricacy of formulation design presents a significant challenge, as SRNPs necessitate a harmonious integration of material selection, drug loading, and stimulus-responsive mechanisms to accommodate the fluctuations of the hypoxic tumor microenvironment. Moreover, scalability and production consistency represent significant challenges. Laboratory-scale production processes may encounter difficulties in ensuring consistent quality and performance of nanoparticles during industrialization, particularly for multifunctional designed SRNPs, where fabrication complexity is heightened [206].

The biodistribution and targeted therapeutic efficacy of SRNPs are essential for in vivo performance. The variability of hypoxic tumors, biological obstacles, and individual patient differences may influence the accurate administration of SRNPs. Despite nanoparticles’ ability to induce drug release via specific stimuli, attaining accurate tumor localization and profound penetration continues to pose a challenge. Moreover, pharmacokinetic factors, including blood stability, clearance rate, and accumulation in non-target tissues, must be optimized to guarantee therapeutic efficacy and safety.

Safety is a fundamental consideration for clinical applications. While SRNPs are engineered to react to particular stimuli within tumors, they may induce off-target effects in healthy tissues, resulting in systemic toxicity or immune responses. Moreover, the potential bioaccumulation of nanoparticles and their long-term impacts on organs (e.g., liver, spleen) have not been sufficiently studied and necessitate further toxicological and safety assessment. These factors impose increased requirements for the advancement of the clinical application of SRNPs [207].

To surmount the aforementioned challenges, it is necessary to further optimize SRNP design through the implementation of personalized medicine and combination therapy strategies in the future. For instance, treatment regimens can be customized to align with the unique characteristics of a patient’s tumor. Additionally, the combination of SRNPs with modalities such as radiotherapy, chemotherapy, and immunotherapy holds promise in enhancing overall efficacy and countering drug resistance. The employment of advanced manufacturing technologies, including microfluidics and 3D printing, holds promise in enhancing the precision and consistency of the production process. The translation of SRNPs into clinical practice for the treatment of hypoxic tumors can be accelerated by interdisciplinary collaboration, technological innovation, and regulatory partnerships, ensuring the provision of safer and more effective solutions.

### 5.3. Toxicity and Safety of Polymeric Nanomedicines

Nanomedicine has significantly enhanced therapeutic outcomes by leveraging nanotechnology to improve drug delivery while reducing toxicity to normal tissues. However, the safety and toxicity concerns of polymeric nanomedicines remain a critical research focus in clinical translation. Although these nanomedicines can increase intratumoral drug concentration through the enhanced permeability and retention (EPR) effect, their inconsistent performance across diverse tumor models poses challenges for clinical application [208]. Additionally, certain polymer-based nanomedicines may induce non-specific cytotoxicity, adversely affecting healthy tissues [15]. For example, some polymers are recognized and phagocytosed by macrophages due to their surface properties, thereby reducing tumor accumulation [209]. The biocompatibility issues of polymeric nanomedicines primarily stem from their interactions with biological systems; many polymers may trigger immune responses in the bloodstream, leading to rapid clearance or toxic side effects.

To address safety concerns, studies suggest that optimizing design parameters can reduce systemic toxicity and enhance targeting efficacy. For instance, a novel polymeric prodrug system with charge-switching functionality demonstrated superior tumor-targeting ability, effectively suppressing tumor proliferation while minimizing damage to normal cells [210]. Additionally, bioengineering strategies—such as coating drug-loaded polymeric microcapsules with bacterial outer membrane vesicles—have been shown to activate host immune responses for more effective cancer immunotherapy with reduced adverse effects [211]. Emerging polymers like amino acid-derived variants exhibit excellent biocompatibility and enable stimulus-responsive targeted drug release, further improving anti-tumor efficacy [4].

### 5.4. Research on Specific Applicability to Oral Cancer Is Still Pending

Although studies have shown that hypoxia plays an important role in oral cancer progression and treatment resistance, many of the key mechanisms associated with this phenomenon remain underexplored [212]. Firstly, the regulation of the tumor immune microenvironment by hypoxia remains a significant unresolved question. While it is established that hypoxia inhibits the immune response, its precise impacts on immune cell infiltration and immune evasion remain unclear [213]. For example, the mechanisms by which hypoxia alters the function of immune cells, such as T cells, macrophages, and dendritic cells, and how these cells interact via cytokines and signaling pathways still require further investigation [214]. Furthermore, while PD-L1 is implicated in hypoxia-induced immune evasion, its precise association with immunosuppressive cell populations, including regulatory T cells and myeloid-derived suppressor cells, requires further clarification [215].

Secondly, the effect of hypoxia on oral cancer stem cells is also unknown. Cancer stem cells are thought to be at the root of tumor recurrence and metastasis, and hypoxia may affect their maintenance and proliferation by altering signaling pathways (e.g., Notch, Wnt, and Hedgehog) [216]. Nonetheless, research remains limited regarding the manner in which hypoxia augments cancer stem cell characteristics (such as self-renewal and differentiation potential) and the implications of these alterations on treatment efficacy. The significance of hypoxia-induced metabolic reprogramming in oral cancer requires urgent elucidation. The mechanisms by which hypoxia induces the transition from oxidative phosphorylation to glycolysis (Warburg effect) in tumor cells, and how this metabolic alteration enhances aggressiveness, drug resistance, and relapse, remain pivotal inquiries in research [217].

Furthermore, the role of hypoxia in oral cancer treatment resistance remains a critical research focus. Hypoxia may enhance tumor cell resistance to chemotherapy, radiotherapy, and targeted therapies by modulating signaling pathways (e.g., PI3K/Akt, MAPK, and NF-κB) while altering the tumor microenvironment to reduce drug permeability or facilitate immune escape [218]. However, the specific modes of regulation of these mechanisms require further validation. Equally important, the impact of hypoxia-induced angiogenesis on tumor heterogeneity and therapeutic efficacy has not been fully elucidated. Structural defects in the neovasculature may further exacerbate tumor hypoxia, affecting the efficiency of drug and oxygen delivery and thus leading to therapeutic failure [219].

There are five main areas where research on hypoxia in oral cancer is lacking: immune interactions, metabolic reprogramming, angiogenesis, therapeutic resistance, and cancer stem cell maintenance [217]. Filling these knowledge gaps will not only deepen our understanding of oral cancer pathogenesis, but also offer the possibility of developing more precise and efficient therapeutic strategies, bringing new hope for improving patient prognosis [220].

## 6. Conclusions and Outlook

### 6.1. Summary of the Potential for Stimuli-Responsive Polymer Nanomedicines

Hypoxic tumors present a significant obstacle to traditional therapies like chemotherapy and radiotherapy because of their diminished oxygen levels and modified metabolism. Stimuli-responsive polymeric nanomedicines provide an effective and safe approach for precision therapy by reacting to the distinct characteristics of the hypoxic microenvironment. These nanomedicines can selectively release drugs in hypoxic areas via hypoxia-sensitive mechanisms (e.g., nitroimidazole linkers or azobonds), minimizing effects on healthy tissues while markedly enhancing the efficacy of oncology therapies.

Moreover, a hypoxic microenvironment diminishes the efficacy of oxygen-dependent treatments like radiotherapy, while oxygen-carrying nanoparticles (e.g., perfluorocarbon nanoparticles) and oxygen-releasing nanoparticles markedly enhance the sensitivity of radiotherapy and chemotherapy by augmenting the oxygenation levels in the tumor region. These nanomedicines can induce the production of ROS to eliminate tumor cells by leveraging the redox imbalance in the hypoxic microenvironment, thus augmenting cytotoxicity and therapeutic effectiveness.

The multifunctional design of stimuli-responsive polymeric nanodrugs, incorporating a dual response to hypoxia and acidic pH, enhances the precision of drug release and therapeutic efficacy. These drug platforms can transport various therapeutic agents (e.g., hypoxia-activated prodrugs and radiotherapy sensitizers) to produce synergistic effects via combination therapies, which are especially effective for drug-resistant or treatment-refractory tumors.

By surmounting the therapeutic challenges presented by the hypoxic microenvironment, these novel designs exhibit the capacity to enhance cancer treatment, both by increasing efficacy and by minimizing side effects. Advancements in technology and ongoing research suggest that stimuli-responsive polymeric nanomedicines will emerge as a crucial instrument for treating hypoxic tumors, resulting in improved prognoses and treatment alternatives for patients with solid tumors.

### 6.2. Prospective Forecasts in the Field of Treatment of Hypoxic Tumors and Oral Cancer

SRPNs exhibit significant promise in the management of hypoxic tumors and oral carcinoma. The distinctive characteristics of the TME, including hypoxia, acidic pH, and alterations in enzyme activity, present opportunities for SRPNs to optimize drug delivery and improve therapeutic efficacy. The capacity of these nanomedicines to facilitate targeted release in reaction to particular stimuli offers innovative solutions to address the constraints of traditional therapies.

The management of oral cancer is frequently constrained by its intricate microenvironmental features, notably pronounced hypoxic conditions. SRPNs can be engineered to react to hypoxic environments by selectively delivering the drug to the tumor site, thereby ensuring highly effective localized treatment. This targeted delivery technology markedly enhances drug concentration in the tumor while reducing side effects in healthy tissue. Moreover, SRPNs can acclimatize to the acidic and hypoxic milieu of the tumor and improve drug permeation across biological barriers like the mucosa, thereby augmenting the therapeutic efficacy of oral cancer treatment.

Hypoxia is frequently linked to therapeutic resistance, and SRPNs can address this issue by adapting to the tumor’s hypoxic microenvironment, pH, or enzymatic activity. These nanomedicines can precisely release drugs at the targeted site, effectively overcoming the resistance posed by the tumor microenvironment. The selective release characteristic of SRPNs diminishes systemic toxicity and adverse effects associated with conventional therapies, making them particularly appropriate for oral cancer patients in need of localized treatment, thereby enhancing both efficacy and safety.

The multifunctionality of SRPNs enables their integration with various therapeutic modalities, including chemotherapy, immunotherapy, and photothermal therapy, thereby creating new opportunities for combination therapies. SRPNs possess a multi-stimulus response capability, allowing for the incorporation of various therapeutic agents within a single nanocarrier to attain synergistic therapeutic effects. Moreover, SRPNs can be customized to align with the distinct attributes of a patient’s tumor microenvironment, offering a potent instrument for personalized cancer treatment. Subsequent research anticipates that SRPNs will evolve into a secure, effective, and precise therapeutic platform, enhancing treatment outcomes and quality of life for oral cancer patients.

### 6.3. Suggested Directions for Future Research

#### 6.3.1. The Need for Interdisciplinary Co-Operation

SRPNs exhibit significant promise in the management of hypoxic tumors and oral cancer. The distinctive characteristics of the TME, including hypoxia, acidic pH, and alterations in enzyme activity, present opportunities for SRPNs to optimize drug delivery and improve therapeutic efficacy. The capacity of these nanomedicines to facilitate targeted release in reaction to particular stimuli offers innovative solutions to address the constraints of traditional therapies.

The management of oral cancer is frequently constrained by its intricate microenvironmental features, notably pronounced hypoxic conditions. SRPNs can be engineered to react to hypoxic environments by specifically delivering the drug to the tumor site, thereby guaranteeing highly effective localized treatment. This targeted delivery technology markedly enhances drug concentration in the tumor while reducing side effects in healthy tissue. Moreover, SRPNs can acclimatize to the acidic and hypoxic milieu of the tumor and improve drug permeation across biological barriers like the mucosa, thereby augmenting the therapeutic efficacy of oral cancer treatment.

Hypoxia is frequently linked to therapeutic resistance, and SRPNs can address this resistance by adapting to the tumor’s hypoxic environment, pH levels, or enzymatic activity. These nanomedicines can release drugs precisely at the targeted location, effectively overcoming the resistance barrier posed by the tumor microenvironment. The selective release characteristic of SRPNs diminishes systemic toxicity and side effects associated with conventional therapies, making them particularly appropriate for oral cancer patients needing localized treatment, thereby enhancing both efficacy and safety.

The multifunctionality of SRPNs enables their integration with various therapeutic modalities, including chemotherapy, immunotherapy, and photothermal therapy, thereby creating new opportunities for combination therapies. SRPNs possess a multi-stimulus response capability, allowing for the incorporation of various therapeutic agents within a single nanocarrier to attain synergistic therapeutic effects. Moreover, SRPNs can be tailored to the distinct attributes of a patient’s tumor microenvironment, offering a potent instrument for personalized cancer treatment. Further research anticipates that SRPNs will evolve into a safe, efficient, and precise therapeutic platform, enhancing therapeutic outcomes and quality of life for oral cancer patients.

#### 6.3.2. Augmented Emphasis on Clinical Translational Research

SRPNs exhibit significant promise in the treatment of hypoxic tumors and oral cancer; however, their transition from laboratory research to clinical applications necessitates the resolution of several critical challenges. Primarily, biocompatibility and biodegradability are essential for ensuring safety. These nanoparticles must circulate in the body and be capable of degrading into non-toxic by-products following drug release. Surface modification techniques, including the application of hydrophilic coatings or specific ligands, can enhance in vivo half-life and optimize drug delivery efficiency. Moreover, the optimization of drug loading and release kinetics is crucial for effective therapy, particularly in hypoxic tumor microenvironments where precise release control is necessary.

Enhancing the targeting and specificity of SRPNs is essential for attaining effective therapies. By optimizing particle size, charge, and surface modifications, SRPNs can more accurately identify and target distinct characteristics of the tumor microenvironment, including hypoxia, acidity, or elevated expression of specific receptors. Moreover, integrating SRPNs with additional therapeutic modalities, including chemotherapy, immunotherapy, or photothermal therapy, can further augment the therapeutic efficacy. These combination therapies aim to address the issues of resistance and ineffectiveness encountered by conventional treatments in hypoxic tumors, while reducing toxicity to normal tissues.

Ensuring the safety and viability of SRPNs necessitates thorough toxicological evaluations and pharmacokinetic analyses. Research must elucidate the metabolic pathways, distribution characteristics, and clearance mechanisms of these nanoparticles in vivo to optimize dosage and treatment protocols. The utilization of animal models that replicate the human tumor microenvironment in preclinical studies will enhance predictability and facilitate more successful clinical trials. Moreover, the standardization of extensive manufacturing processes and adherence to GMP-compliant quality control are crucial for clinical applications, as these elements are essential to guarantee the high purity, stability, and reproducibility of nanoparticles.

Customized therapy and stringent regulatory supervision will be pivotal in future clinical translation. The design of SRPNs can be customized to enhance drug loading and release mechanisms for various patient tumor types and microenvironmental attributes. Clinical trials must prioritize patients’ quality of life, treatment tolerability, and potential adverse effects to guarantee efficacy and safety. Simultaneously, researchers must adhere to regulatory mandates from pharmaceutical authorities and consider ethical and environmental implications during production and application. Emerging technologies like AI-driven drug formulation and personalized medicine could significantly impact the development of polymeric nanomedicines, enabling more precise and effective treatments by optimizing drug design and delivery based on individual patient needs. Through interdisciplinary collaboration and ongoing innovation, SRPNs are anticipated to serve as an effective modality for treating hypoxic tumors and oral cancers, resulting in more precise and efficient therapeutic options for patients.

In summary, several key steps are necessary to bring these nanomedicines into clinical use. Firstly, rigorous preclinical testing is required to fully assess the safety and efficacy of SRPNs in relevant animal models, particularly those that mimic the human tumor microenvironment. This includes comprehensive toxicological evaluations and pharmacokinetic studies to understand the metabolic pathways, distribution characteristics, and clearance mechanisms of the nanoparticles. Secondly, the development of standardized manufacturing processes and adherence to Good Manufacturing Practices (GMPs)—compliant quality control are crucial to ensure the high purity, stability, and reproducibility of the nanoparticles for clinical applications. Thirdly, successful translation to clinical trials will depend on careful design and execution, prioritizing patients’ quality of life, treatment tolerability, and potential adverse effects. Clinical trials must be conducted in accordance with regulatory mandates from pharmaceutical authorities, ensuring ethical and environmental considerations are met. Finally, the integration of emerging technologies, such as AI-driven drug formulation and personalized medicine, could further optimize the design and delivery of SRPNs, enabling more precise and effective treatments based on individual patient needs. These advancements will help to overcome the challenges associated with hypoxic tumors and improve the prognosis for patients with oral cancer.

## Figures and Tables

**Table 1:** Summary of cited examples of nanoagents designed for cancer treatment.

Nanomedicine	Response Type	Drug Release/Efficacy (Compared to Control Group)	Target Protein	Innovations	Reference
cHDEA	pH	3 times	— *^a^*	Reversible drug delivery	[21]
iCPDN	pH	5 times	HIF-1α	Overcoming hypoxia-induced chemotherapy resistance	[22]
NP@PEDOX/PSP	PDT	3.3 times	— *^a^*	Imaging-guided photodynamic immunotherapy	[23]
PTX-PLGA-gel-MS	pH	1.5 times	— *^a^*	Sustained rapid-release drugs	[24]
TOS-Ace-PEG-Bio	pH	2.87 times	CD44 receptor	Rapid drug release and high safety	[25]
DDMSNs-DOX HA	Redox/pH	2 times	— *^a^*	High drug-loading capacity Good degradability	[26]
DSNs	Redox	5 times	— *^a^*	Controlled release of drugs to reduce side effects	[27]
SynB3-PVGLIG-PTX	Enzyme	2.27 times	MMP-2	MMP-2 targets high permeability with low side effects	[28]
PEG-b-PCPTEMA	ROS	16 times	— *^a^*	Active modulation of the tumor microenvironment	[29]
LDGI	PDT	6.8 times	— *^a^*	Controlled drug release Photothermal therapy	[30]
SOR-NPBDP/PTX	ROS/PDT	2.5 times	— *^a^*	Combined photodynamic chemotherapy	[31]
MCNC	Magnetic	3.5 times	— *^a^*	Spatiotemporal control of drug release	[32]
DOX/Br NP2	pH	1.39 times	— *^a^*	Improve drug penetration and increase drug concentration	[33]
DOX-STB	pH	2.5 times	— *^a^*	Highly controlled and sustained release drugs	[34]
Mo154Gel	pH/PDT	2.27 times	— *^a^*	Self-healing injectable hydrogel	[35]
A2-APM	redox response	2.93 times	PDL-1	Modulating the immunosuppressive tumor microenvironment	[36]
DOX-HR-LPs	Hypoxia	4 times	— *^a^*	Enhances anti-tumor efficacy and reduces systemic toxicity	[37]
HR-NPs	Hypoxia	2 times	— *^a^*	Reduced systemic toxicity and improved biodistribution facilitated tumor accumulation of nanoparticles	[38]
ALN-HR-PMs/DOX	Hypoxia	4 times	— *^a^*	Inhibition of osteoclast activity and promotion of osteoblast activity effectively inhibit tumor growth and protect bone structure.	[39]
GLU-PTX	Hypoxia	2 times	CA IX	Exhibits good stability under physiological conditions and rapidly releases the active drug in hypoxic environments	[40]
H-MnO2-PEG/TP	Hypoxia	— *^a^*	HIF-1α	Relief of tumor hypoxia and inhibition of angiogenesis	[41]
PPGN@DOX	Hypoxia	— *^a^*	— *^a^*	Effectively internalizes into the nucleus of the tumor cell and provides superior anti-tumor activity	[42]
D@HRGF	Hypoxia	2.1 times	Folate receptor FR	Low toxicity and good biocompatibility	[43]
NP-PDT@Reg	PDT	4 times	— *^a^*	Normalizing blood vessels to overcome tumor hypoxia and reprogramming macrophages	[44]
Cu-PPT NPs	PDT	— *^a^*	— *^a^*	Sustained stimulation of systemic anti-tumor immune responses and reactivation of T cells	[45]
DOX-HR-LP	Hypoxia	3 times	— *^a^*	Significantly improves anti-tumor efficacy and significantly reduces DOX toxicity	[37]
PA/HA-Ce6@TPZ NPs	PDT/Hypoxi	6 times	— *^a^*	More precise control of efficacy and toxicity of chemotherapy drugs	[46]
Polymer–drug conjugate (PDC)	Enzyme	— *^a^*	Carboxylesterase hCE2	Improving the efficacy of anticancer drugs and reducing systemic toxicity	[47]
HRM NPs	Hypoxia	6 times	Nitroreductase (NTR)	Modulation of the redox state of tumor cells enhances the efficiency of chemotherapeutic drug release	[48]
PCL@GSK-diABZI/aPD-1	pH	— *^a^*	PD-1 MMP-2	Creating a positive feedback loop in the cancer immune cycle to enhance anti-tumor immune response	[49]

*^a^* Not covered in the original article.

## Data Availability

The data from this study are available from the corresponding author upon request, due to privacy reasons.

## Abbreviations

HIF	hypoxia-inducible factor
MMPs	matrix metalloproteinases
VEGF	vascular endothelial growth factor
PLGA	poly (lactic acid-glycolic acid)
EPR	enhanced permeability and retention
PLA	polylactic acid
PEG	polyethylene glycol
HA	hyaluronic acid
DOX	doxorubicin
NO	nitric oxide
ROS	reactive oxygen species
NIR-II	near-infrared II
PACT	photoacoustic computed tomography
WHO	World Health Organization
PLL	polylysine
MTX	methotrexate
HIFs	hypoxia-inducible factors
TNBC	triple-negative breast cancer
TME	tumor microenvironment
shHIF-1α	ShRNA targeting HIF-1α
MIPs	molecularly imprinted polymer nanoparticles
QDs	quantum dots
CAIX	carbonic anhydrase IX
PEG-b-PDMT	poly (ethylene glycol)-b-poly (dithiothiol methacrylate)
PTT	photothermal therapy
DSN	DiR-loaded self-assembled nanoparticles
NPs	nanoparticles
MSCs	mesenchymal stem cells
SOR-NP	single-linear oxygen-responsive nanoparticles
BDP	dipyrrolylmethylene boron
PDT	photodynamic therapy
Br	bromelain
STBs	shear-thinning biomaterials
BBB	blood–brain barrier
RNAi	RNA interference
HIF-1	hypoxia-inducible factor 1
HR-NPs	hypoxia-responsive polymer nanoparticles
TMEs	tumor microenvironments
CESs	carboxylesterases
CYPs	cytochrome P450 enzymes
pNGs	peptide-crosslinked nanogels
HRM NPs	hypoxia-responsive micelle nanoparticles
PAP NPs	PTX prodrug nanoparticle
HAPs	hypoxia-activated prodrugs
EGFR	Epidermal Growth Factor Receptor
SRPNs	stimuli-responsive polymer nanomedicines
ATLP	acid-triggered ligand presentation
iPDOX	IRGD-modified adriamycin polymer prodrug
NIR	near-infrared
ROS	reactive oxygen species
LA	lactobionic acid
OA	oleic acid
mPEG	methoxy poly (ethylene glycol)
CPT	camptothecin
SPNs	stimuli-responsive polymeric nanoparticles
PDT	photodynamic therapy
GMP	Good Manufacturing Practices
PDC	polymer–drug conjugate
NTR	nitroreductase
